# Towards a scientific interpretation of the *terroir* concept: plasticity of the grape berry metabolome

**DOI:** 10.1186/s12870-015-0584-4

**Published:** 2015-08-07

**Authors:** Andrea Anesi, Matteo Stocchero, Silvia Dal Santo, Mauro Commisso, Sara Zenoni, Stefania Ceoldo, Giovanni Battista Tornielli, Tracey E. Siebert, Markus Herderich, Mario Pezzotti, Flavia Guzzo

**Affiliations:** Biotechnology Departement, University of Verona, Strada le Grazie 15, 37134 Verona, Italy; S-IN Soluzioni Informatiche, Via G. Ferrari 14, 36100 Vicenza, Italy; The Australian Wine Research Institute, P.O. Box 197, Glen Osmond, SA 5064 Adelaide, Australia; Present address: Physics Department, Laboratory of Bioorganic Chemistry, University of Trento, Via Sommarive 14, 38123 Trento, Italy

## Abstract

**Background:**

The definition of the *terroir* concept is one of the most debated issues in oenology and viticulture. The dynamic interaction among diverse factors including the environment, the grapevine plant and the imposed viticultural techniques means that the wine produced in a given *terroir* is unique. However, there is an increasing interest to define and quantify the contribution of individual factors to a specific *terroir* objectively. Here, we characterized the metabolome and transcriptome of berries from a single clone of the Corvina variety cultivated in seven different vineyards, located in three macrozones, over a 3-year trial period.

**Results:**

To overcome the anticipated strong vintage effect, we developed statistical tools that allowed us to identify distinct *terroir* signatures in the metabolic composition of berries from each macrozone, and from different vineyards within each macrozone. We also identified non-volatile and volatile components of the metabolome which are more plastic and therefore respond differently to *terroir* diversity. We observed some relationships between the plasticity of the metabolome and transcriptome, allowing a multifaceted scientific interpretation of the *terroir* concept.

**Conclusions:**

Our experiments with a single Corvina clone in different vineyards have revealed the existence of a clear *terroir*-specific effect on the transcriptome and metabolome which persists over several vintages and allows each vineyard to be characterized by the unique profile of specific metabolites.

**Electronic supplementary material:**

The online version of this article (doi:10.1186/s12870-015-0584-4) contains supplementary material, which is available to authorized users.

## Background

Wine is a complex mixture of metabolites derived from grape berries, yeasts and bacteria during fermentation, and for barrel-aged wine, also the oak and other woods used for cask making [1]. The chemical reactions that occur during vinification can further transform grape and yeast metabolites, and the ageing process increases this complexity. Because grapes provide the basis for many wine aromas, flavors and colors, there is much interest in factors affecting the composition of ripe berries [1–3].

The metabolites found in grapes fall into two main groups: volatile and non-volatile compounds, present mainly in the berry skin and flesh. Volatile organic compounds (VOCs) are low-molecular-weight aldehydes, ketones, alcohols, esters, lactones, terpenes, norisoprenoids, methoxypyrazines and thiols (usually less than 300 Da), which vaporize rapidly at room temperature. Non-volatile compounds include a diverse range of primary and secondary metabolites. Sugars (mainly glucose and fructose), organic acids (predominantly tartaric and malic acid) and amino acids (mostly proline and arginine) are the important primary metabolites, mainly present in the berry flesh. Most of the secondary metabolites are phenylpropanoids, e.g., anthocyanins, flavonoids, phenolic acids, flavan-3-ols, procyanidins, polymeric tannins, stilbenes and viniferins, which are typically found predominantly in the berry skin. All these compounds have been widely studied because they affect wine quality and are thought to be beneficial for human health [3–7].

In oenology, the environmental factors that characterize a specific vineyard and impact grape and wine quality are known as terroir. Seguin [8] defined *terroir* as an interactive ecosystem, in a given place, including climate, soil and the vine (cultivar and rootstock). In a non-scientific context, the concept of *terroir* implies that a wine produced in a given region is unique and cannot be reproduced elsewhere even if the grape and winemaking techniques are painstakingly duplicated. The importance of *terroir* on grape and wine quality is the subject of debate, particularly because of its commercial and marketing relevance.

In terms of biology, *terroir* is reflected in the differences in fruit composition caused by growing the vine in a different environment, given that the accumulation of berry metabolites is influenced by communication between the vine and its biotic and abiotic surroundings. Determining the objective impact of a specific *terroir* is challenging because many factors and their interactions may be involved, including climate, soil, topography, vineyard characteristics, cultivar, vine water status, rootstock and viticultural practices. Previous investigations have focused on single environmental factors such as specific forms of abiotic stress, and have identified positive correlations with the expression of certain genes and the abundance of specific metabolites [9–11].

Recently, the metabolomics approaches have been successfully used to discriminate Pinot noir grapes (and related wines) from two different but close vineyards, referred as terroirs, managed by the same vine grower, to reduce the impact of the human intervention [12, 13]. Here we used an opposite approach to characterize the terroir effect on berry composition, given that human intervention is considered one of the components of terroir. On the other side, we removed as much complexity as possible by working not only on a single cultivar but also on a single clone, thus eliminating much possible variability due to genetic background. This aspect has been overlooked in previous studies of the terroir concept, even though clonal selection is widely practiced in viticulture, suggesting that somatic modification has a significant effect on berry and wine quality.

We used an untargeted metabolomics approach based on liquid chromatography–mass spectrometry (LC-MS) and gas chromatography–mass spectrometry (GC-MS) to investigate the effects of *terroir* on a single clone *of Vitis vinifera* cv. Corvina (clone 48) in seven different vineyards managed by distinct vine growers and located in three different macrozones, over a 3-year trial period. We previously used the same experimental conditions to define the plasticity of the grapevine berry transcriptome, revealing that 5 % of the Corvina transcriptome is used for *terroir*-specific adaptation [14]. We found that the phenylpropanoid pathway, especially resveratrol biosynthesis, was one of the most environmentally-dependent metabolic components, with a good correlation between metabolite levels and the induction of gene expression [14].

Here, we anticipated a strong vintage-specific effect on the berry metabolome and therefore developed statistical tools to overcome this effect, allowing us to explore the metabolomic and transcriptomic data in detail. Even when the vintage effect was dominant, the three macrozones showed distinct *terroir*-specific signatures in fruit composition, and berries from each individual vineyard within the macrozone were characterized by specific chemical traits. We conclude that different components of the metabolome and transcriptome can respond to unique interactions of factors within each *terroir*.

## Methods

### Plant material

*Vitis vinifera* cv. Corvina clone 48 berries were sampled during the 2006, 2007 and 2008 growing seasons at three time points, corresponding to véraison, mid-ripening and the putative full-mature stage, in seven commercial vineyards located in three different macrozones (Lake Garda, Valpolicella and Soave) in the province of Verona, Italy. Fully mature berries were harvested in all vineyards on the same days: 18 September 2006, 29 August 2007, and 23 September 2008. Berries at véraison were collected in all the vineyards on 8 August 2006, 18 July 2007, 12 August 2008, while pre-ripening grape was harvested on 4 September 2006, 8 August 2007, 2 September 2008.

The principal features of each vineyard are summarized in Additional file 1: Table S1, and major meteorological data over the 3-year sampling period are reported in Additional file 2: Table S2. For each of the accessions (producer/year), we harvested 30 clusters from different positions along two vine rows, with randomized heights and locations on the plant. Three berries were selected randomly from each cluster, avoiding those with visible damage and/or signs of infection. Then we repeated the sampling procedure three times to obtain three separated pools. The berries were frozen immediately in liquid nitrogen. Just before metabolite extraction and microarray analysis, 10 frozen berries from each pool were crushed and finely ground after removing the seeds. The representativeness of these powdered pools was preliminarily assessed by HPLC-ESI-MS analysis and visual inspections of the resulting chromatograms (not shown).

### Enological analyses

Three replicates of 20 berry samples were crushed and the resulting must was clarified by centrifugation. The clarified matrix was used for pH and reducing sugars measurements. Reducing sugars were quantified enzymatically using a commercial kit (Glucose/Fructose Kit, Enologica Vason S.p.a., Italy), following the instructions manual.

### Extraction, analysis and identification of non-volatile metabolites

LC-MS-grade acetonitrile, formic acid and water, and HPLC-grade methanol, were purchased from Sigma-Aldrich (St. Louis, MO, USA). Unisolv-grade *n*-pentane and Suprasolv-grade ethylacetate were purchased by Merck (Darmstadt, Germany).

The metabolites were extracted at room temperature in three volumes (w/v) of methanol acidified with 0.1 % (v/v) formic acid in an ultrasonic bath (Falc Instruments, Bergamo, Italy) at 40 kHz for 15 min. Extracts were centrifuged twice for 10 min at 16,000 × *g* at 4 °C, diluted 1:2 (v/v) in milliQ water and passed through 0.2-μm Sartorius Minisart RC4 filters (Sartorius-Stedim Biotech, GmbH, Goettingen, Germany).

The HPLC-ESI-MS system comprised a Beckman Coulter Gold 127 HPLC (Beckman Coulter, Fullerton, CA) equipped with a System Gold 508 Beckman Coulter autosampler. Metabolites were separated on an analytical Alltima HP RP-C18 column (150 × 2.1 mm, particle size 3 μm) equipped with a C18 guard column (7.5 × 2.1 mm) both purchased from Alltech Associates Inc. (Derfield, IL, USA), using mixtures of solvent A (5 % (v/v) acetonitrile, 0.5 % (v/v) formic acid in water) and solvent B (100 % acetonitrile). A linear gradient, at a constant flow rate of 0.2 ml/min, was established from 0 to 10 % B in 5 min, from 10 to 20 % B in 20 min, from 20 to 25 % B in 5 min, and from 25 to 70 % B in 15 min. Each sample was analyzed in duplicate, with a 30-μl injection volume and 20 min re-equilibration between each analysis.

Mass spectra were acquired using a Bruker Esquire 6000 ion trap mass spectrometer (Bruker Daltonik GmbH, Bremen, Germany) equipped with an electrospray ionization source. Alternate negative and positive ion spectra were recorded in the range 50–1500 *m/z* (full scan mode, 13,000 *m/z* per second). For metabolite identification, MS/MS and MS^3^ spectra were recorded in negative or positive mode in the range 50–1500 *m/z* with fragmentation amplitude of 1 V. Nitrogen was used as the nebulizing gas (50 psi, 350 °C) and drying gas (10 l/min). Helium was used as the collision gas. The vacuum pressure was 1.4 × 10^−5^ mbar. parameters: capillary source +4000 V; end plate offset –500 V; skimmer –40 V; cap exit –121 V; Oct 1 DC –12 V; Oct 2 DC –1.70 V; lens 1 5 V; lens 2 60 V; ICC for positive ionization mode 20,000; ICC for negative ionization mode 7000.

MS data were collected using the Bruker Daltonics Esquire v5.2 and Esquire Control v5.2 software, and processed using the Bruker Daltonics Esquire v5.2 and Data Analysis v3.2 software (Bruker Daltonik GmbH, Bremen, Germany). Metabolites were identified by comparing the *m/z* values, fragmentation patterns (MS/MS and MS^3^) and retention times of each signal with those of available commercial standards and or with our previously published data [15, 16]. When commercial standards were not available, fragmentation patterns were also compared with those published in the literature or on-line databases such as MassBank (www.massbank.jp/en/database.html) and Human Metabolome Database (http://www.hmdb.ca/). HPLC-diode array detector (DAD) analysis was carried out using a Beckman Coulter Gold 126 Solvent Module equipped with Gold 168 Diode Array Detector under the same analytical conditions described above, in the wavelength range 190–600 nm. Chromatographic data were collected and processed using Beckman Coulter 32 Karat software v7.0.

### Extraction, analysis and identification of volatile metabolites

Free volatile metabolites were extracted from the same berry samples described above, using three sampling replicates. We transferred 4 g of powdered berry tissue to a 7-ml glass vial with an aluminum insert lid and thawed the tissue for 90 min before extraction. Following the addition of 1 ml MilliQ water and 18.6 μl of a mixture of the internal standards *d*_*13*_*-*hexanol (1000 μg/kg), α-copaene (200 μg/kg) and *d*_*3*_*-*β-ionone (50 μg/kg) dissolved in ethanol (kindly provided by The Australian Wine Research Institute, Adelaide, Australia), the metabolites were extracted with 2 ml of a 1:1 (v/v) mixture of *n*-pentane and ethylacetate, stirred for 10 s, incubated for 15 min in a Branson 3510 ultrasonic bath (Branson Ultrasonic, Danbury, USA) and mixed at room temperature for 2 h on a Rocking Platform Mixer (Ratex Instruments Pty, Boronia, VIC, Australia) at 25 rpm. Liquid extracts were collected and stored in glass vials at –20 °C.

An Agilent Technologies 6890 GC column (Agilent Technologies, Santa Clara, CA, USA) was coupled to an Agilent 5973 N mass-selective detector, each controlled using Agilent G1701CA ChemStation software. The system was also equipped with a Gerstel MPS2 multipurpose sampler controlled by Gerstel Master software v1.81 and a Gerstel CIS-4 cool inlet with twister desorption unit (TDU) fitted with a resilanized borosilicate glass liner with glass wool insert. We cryofocused each 25-μl sample in the Gerstel CIS-4 held at –10 °C and injected the sample in solvent vent mode with an injector temperature of –10 °C. The temperature of the TDU was ramped to 240 °C at 10 °C/s, transferring the trapped metabolites onto the GC column. The TDU was then held at 240 °C for 3 min ensuring no carryover of analytes to the next sample, as confirmed by the analysis of blanks.

The GC was fitted with an Agilent non-polar DB-5MS+ column (60 m × 0.25 mm, 0.25 μm) and the carrier gas was Ultrahigh Purity helium at a linear velocity of 26 cm/s. The initial flow rate was set to 1.0 ml/min in constant-flow mode. The oven temperature was started at 40 °C and held for 7 min before the temperature was increased to 150 °C at 7 °C/min, then to 170 °C at 2 °C/min and then to 240 °C at 20 °C/min and held for 15 min. The MS transfer line was held at 250 °C.

The mass spectrometer quadrupole temperature was set at 150 °C and the source set at 230 °C. Positive ion electron impact spectra at 70 eV were recorded in the *m/z* range 35–350 for scan runs. Selected ion monitoring (SIM) was used for the relative quantification of targeted metabolites. The *n*-alkane series (alkane standard solution C_8_-C_20_, Fluka, Sigma-Aldrich) was run using the same method to benchmark the retention indices. The identity of compounds was verified by comparison with Kovats retention indices and mass spectra with those contained in the NBS, Wiley and AWRI GC-MS databases, and in an “in house” database of spectra of authentic standards. A matching of at least the 90 % was considered for aldehydes, alchools, monoterpenes and C13-norisoprenoids, while for the other metabolites a matching of at least 75 % was used.

### LC/GC-MS data processing

LC-MS chromatograms were transformed into the netCDF format using the Bruker Daltonics Esquire v5.2 and Data Analysis v3.2 software (Bruker Daltonik GmbH, Bremen, Germany).

The open-source software MZmine v2.2 (http://mzmine.sourceforge.net) was used to extract the data, which was processed by median fold change normalization before log transformation and mean centering. The matrix effect did not substantially affect the relative quantification of secondary metabolites under our analytical conditions (data not shown) as we have previously shown [15]. In order to further evaluate the performances of HPLC-ESI-MS for relative quantitation, the HPLC-ESI-MS relative quantitation of the more abundant metabolites were compared with those with obtained by HPLC-DAD, which is a quantitative techniques (Additional file 3).

GC-MS chromatograms were analyzed using Agilent C1701 Chemstation software. Peaks were automatically integrated and the results were checked manually. The data representing 63 samples × 48 identified molecules were normalized by internal standard peak areas to avoid differences in detection efficiencies. Monoterpene and sesquiterpene compounds were normalized to the α-copaene peak area, norisoprenoids to the *d*_3_-β-ionone peak area, and remaining compounds to the *d*_13_-hexanol peak area. The resulting data set was autoscaled before analysis.

### Microarray data

The transcriptomic data from seven out of eleven vineyards sampled in the 2008 growing season (BA, CS, BM, MN, FA, AM and PM) from our previous work [14] were retrieved and reanalyzed in the present work. Briefly, as previously described, the gene expression microarray data were obtained by hybridization to a NimbleGen microarray 090818_Vitus_exp_HX12 (Roche, NimbleGen), which contains probes targeting 29,549 predicted grapevine genes, representing 98.6 % of the genes predicted from the V1 annotation of the 12X grapevine genome (http://srs.ebi.ac.uk/) and 19,091 random probes as negative controls. The expression data were analyzed using T-MeV v4.8.1 software (http://sourceforge.net/projects/mev-tm4/) and were normalized based on the mean center genes/rows adjustment, with Pearson’s correlation metric chosen as the statistical metric. The obtained data set was log-transformed and mean centered prior to analysis.

### Data analysis and modelling

A preliminary data analysis based on ANOVA was performed to highlight the role of vintage and producer on the variation of each single measured metabolite. Since the design of experiments was characterized by restricted randomization because the samples collection resulted to be dependent on the year, we applied a split-plot ANOVA approach where the whole plot factors were the year of sample collection and the replicate while the subplot factor was the producer [17]. This univariate investigation did not take into account the simultaneous relationships among variables but focused solely on the mean and the variance of a single variable. For this reason we applied a suitable multivariate data analysis strategy based on projection methods which allowed us to include the correlation structure among the variables in the modeling of the response of interest.

Exploratory multivariate data analysis was carried out by principal component analysis (PCA) whereas partial least squares projection to latent structures discriminant analysis (PLS-DA), orthogonal projection to latent structures discriminant analysis (O2PLS-DA) and orthogonal constrained PLS-DA (oCPLS2-DA), developed in the present work, were used to investigate differences in the metabolic content of the samples.

Orthogonal constraints can be included in PLS-DA using a suitable orthogonal projection matrix in the maximization problem solved by PLS, as described in Additional file 4. The inclusion of constraints in data modeling allowed us to focus the analysis of the systematic variation of the data based solely on differences between the sample groups, excluding the effects of other factors such as vintage. Indeed, PLS-DA could include the variation related to the vintage in the calculation of the latent space producing models where both “terroir” and vintage confound their effects while oCPLS2-DA is able to generate latent components where the effects of vintage are excluded. In other words, PLS-DA could provide false discoveries depending on the design of the experiment and on the correlation structure of the collected data. For this reason, the year of sample collection was used to build the matrix specifying the constraints obtaining latent structures orthogonal to this metadata by oCPLS2-DA, thus removing information related to the vintage from the data modeling.

Projection methods such as PLS-DA usually produce a large number of latent components compromising a clear interpretation of the model. To focus the structured variation on a suitable space described by a reduced number of latent components, thus simplifying the interpretation of the model, we applied the post-transformation approach described by Dall’Acqua et al. [18]. The weight matrix of the oCPLS2-DA and PLS-DA models were therefore rotated to obtain a new post-transformed model where only *N* – 1 predictive latent components were used to explain the differences between the *N* classes under investigation. The method is described in Additional file 4.

The role played by the measured variables in the models was investigated by suitable correlation loading plots. According to good practice for model building and validation, we performed a permutation test on the class responses and *N*-fold full cross-validation with different values of *N* (*N* = 6, 7, 8) to avoid over-fitting and to evaluate the reliability of the models. The number of latent components was determined on the basis of the first maximum of Q^2^ during 7-fold full cross-validation under the constraint to pass the permutation test on the class responses.

PCA and PLS-DA were carried out using SIMCA v13 (Umetrics, Umea, Sweden) and software platform R v3.0.2 (R Foundation for Statistical Computing) was used to build the oCPLS2-DA model (user-written R function), for post-transforming the models (user-written R function) and for split-plot ANOVA.

In order to investigate the specific response of berry metabolome to terroir specific environmental features, for each of the features listed in Additional file 1: Table S1 several possible classification were created; only some of these combinations resulted in O2PLS-DA models, that were subsequently validated.

## Results

### The fully-mature berry metabolome is principally affected by vintage

Corvina clone 48 berries were harvested at three time points corresponding to the beginning of vèraison (that is the term used by viticulturist to indicate the onset of ripening), mid-ripening and full maturity in seven vineyards located in the three most important macrozones for wine production surrounding Verona (Soave, Valpolicella and Lake Garda; Additional file 1: Table S1) during the 2006, 2007 and 2008 growing seasons. Parameters reflecting the uniform degree of ripeness among different vineyards and growing seasons have been reported in Additional file 1: Table S1 and, only for some of the vineyards/vintages, also in Dal Santo et al., 2013 [14].

HPLC-ESI-MS was used to characterize the non-volatile metabolites. Among 551 signals, 73 were assigned to molecules, 131 to aglycones, fragments and molecular adducts, and the others remained unidentified. The identified metabolites included 18 anthocyanins, 13 flavan-3-ols and procyanidins, 14 flavonols and flavanols, 18 stilbenes and viniferins, 6 hydroxycinnamic acids, and a small number of sugars, amino acids and non-aromatic organic acids. Structural characterization by MS/MS and database searching revealed eight new molecules that were not identified in the previously-reported Corvina metabolome [15, 16]; Additional file 5: Table S3).

GC-MS was used to investigate the volatile molecules, revealing 48 identifiable molecules in the ripe berry metabolome (Additional file 6: Table S4). Many of these molecules were sesquiterpenes (representing 40.8 % of all the compounds identified by GC-MS). The other identifiable volatile compounds were aldehydes (14.3 %), carboxylic acids (12.2 %), monoterpenes (8.2 %), alcohols (8.2 %), hydrocarbons (6.1 %), esters (4.1 %), norisoprenoids (4.1 %) and other sesquiterpenoids (2 %).

The analysis of variance (ANOVA) based on Split-plot design was preliminarly used to retrieve all those metabolites that significantly varied through the different vintages and producers (Additional file 7: Table S5). Considering only the identified metabolites, most of them varied according to the vintage and the producers. Going into details, among the non volatile metabolites, 67 % of them varied according to the vintage and the 69 % according to the producers. These variables belonged to all the main classes of metabolites. Among the volatile metabolites, 39 % of them varied according to the vintage and 67 % of them according to the producers. Interestingly, among the volatile metabolites the sesquiterpenes showed the strongest modulation according to the producers. Then, the effects of vintage and producer on the metabolite profile results to be complex to investigate. For this reason we performed our strategy for data modeling based on orthogonal constrained PLS-DA that allowed us to exclude the effects of vintage on the metabolite profile.

**Fig. 1:**
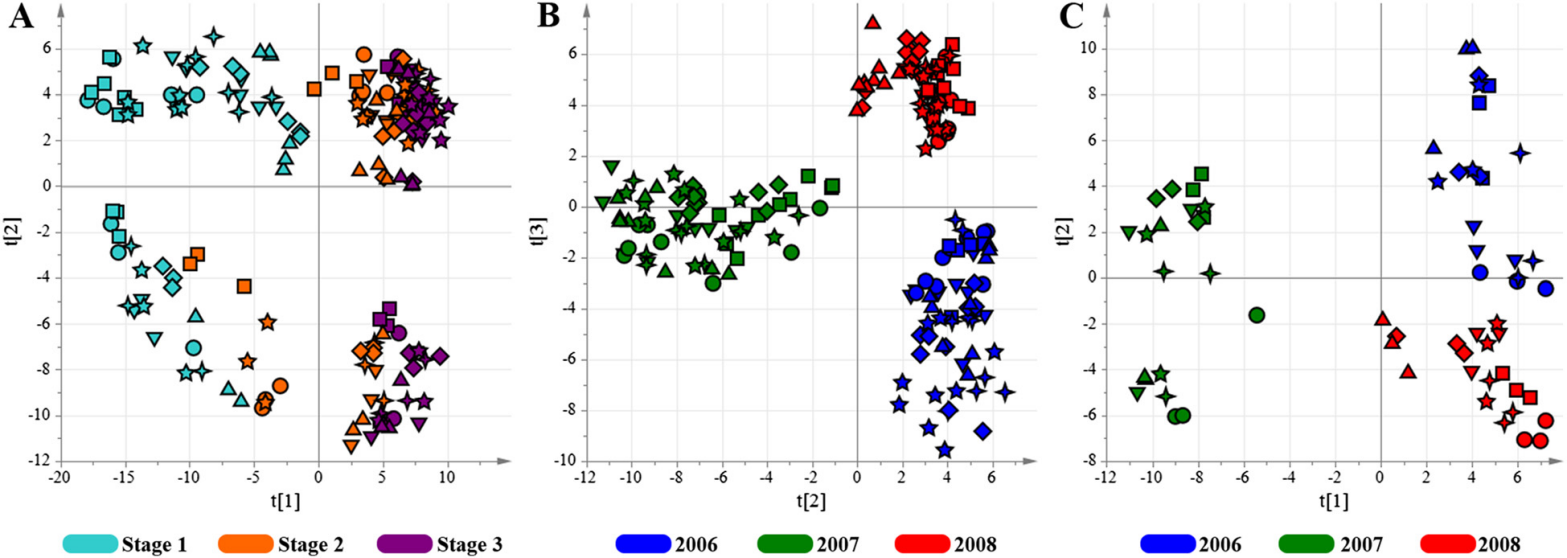
PCA score scatter plot of the model obtained for the metabolites detected by HPLC-ESI-MS. Samples, corresponding to the seven vineyards (sampled in vintages 2006, 2007 and 2008 at three time points) are roughly separated according to developmental stage (**a**; explained variance equal to 44 %). Stage 1: beginning of véraison; stage 2: pre-ripening; stage 3: full maturity. PCA score scatter plot of the same data set used in (**a**) colored according to vintage (**b**; explained variance equal to 20 %). Blue: 2006; green: 2007; red: 2008. PCA score scatter plot of fully-ripe grapes (**c**; explained variance equal to 35 %). Blue: 2006; green: 2007; red: 2008. Vineyards: ▼ = AM; ● = BA; ◼ = BM; ✦ = CS; ♦ = FA; ★ = MN; ▲ = PM

The entire HPLC-ESI-MS data set was explored by PCA. The score scatter plot shows that PC1, explaining 31 % of the total variance, could mainly distinguish the developmental stage, separating véraison stage from mid ripening and fully mature stages (Fig. 1a), whereas PC2 and PC3, explaining 20 % of the total variance, separated the samples according to vintage (Fig. 1b).

By applying a supervised PLS-DA approach, we obtained a reliable model (two latent components, R^2^ = 0.55, Q^2^_6-fold CV_ = 0.51, Q^2^_7-fold CV_ = 0.49, Q^2^_8-fold CV_ = 0.51) showing as expected that the fully mature berry was mainly characterized by higher levels of anthocyanins and stilbenes, and by lower levels of hydroxycinnamic acids and procyanindis, compared to the véraison phase (Additional file 8: Figure S1A, B).

Focusing specifically on fully-mature berries, PCA revealed that the vintage effect was so strong that it prevented any obvious clustering according to vineyards, each representing a specific *terroir* (Fig. 1c). The behavior of the 2006 vintage was intermediate between the 2007 and 2008 vintages, as previously reported for the full transcriptomic data set based on the same biological material [14].

PLS-DA generated a model with two components (R^2^ = 0.93, Q^2^_6-fold CV_ = 0.92, Q^2^_7-fold CV_ = 0.92, Q^2^_8-fold CV_ = 0.91) that could distinguish the vintage. Analysis of the loading structure showed that the 2008 vintage promoted the accumulation of secondary metabolites, particularly anthocyanins and stilbenes (Additional file 8: Figure S1C, D).

The GC-MS data set for fully-mature berries was also investigated by PCA, and showed a rough clustering based on vintage. A clearer separation was obtained by PLS-DA (three components, R^2^ = 0.61, Q^2^_6-fold CV_ = 0.45, Q^2^_7-fold CV_ = 0.51, Q^2^_8-fold CV_ = 0.41) but no metabolites were correlated strongly with a specific vintage (Additional file 8: Figure S1E, F).

### Some metabolome components show enhanced plasticity

The vintage-specific effects on the metabolite content of our berry samples masked the other environmental effects (Fig. 1b, c). We therefore used a constrained technique to model the data, by generating latent variables orthogonal to the vintage by oCPLS2-DA. We initially analyzed the data according to geographical origin (the three macrozones) and then by the different vineyards within each macrozone.

**Fig. 2:**
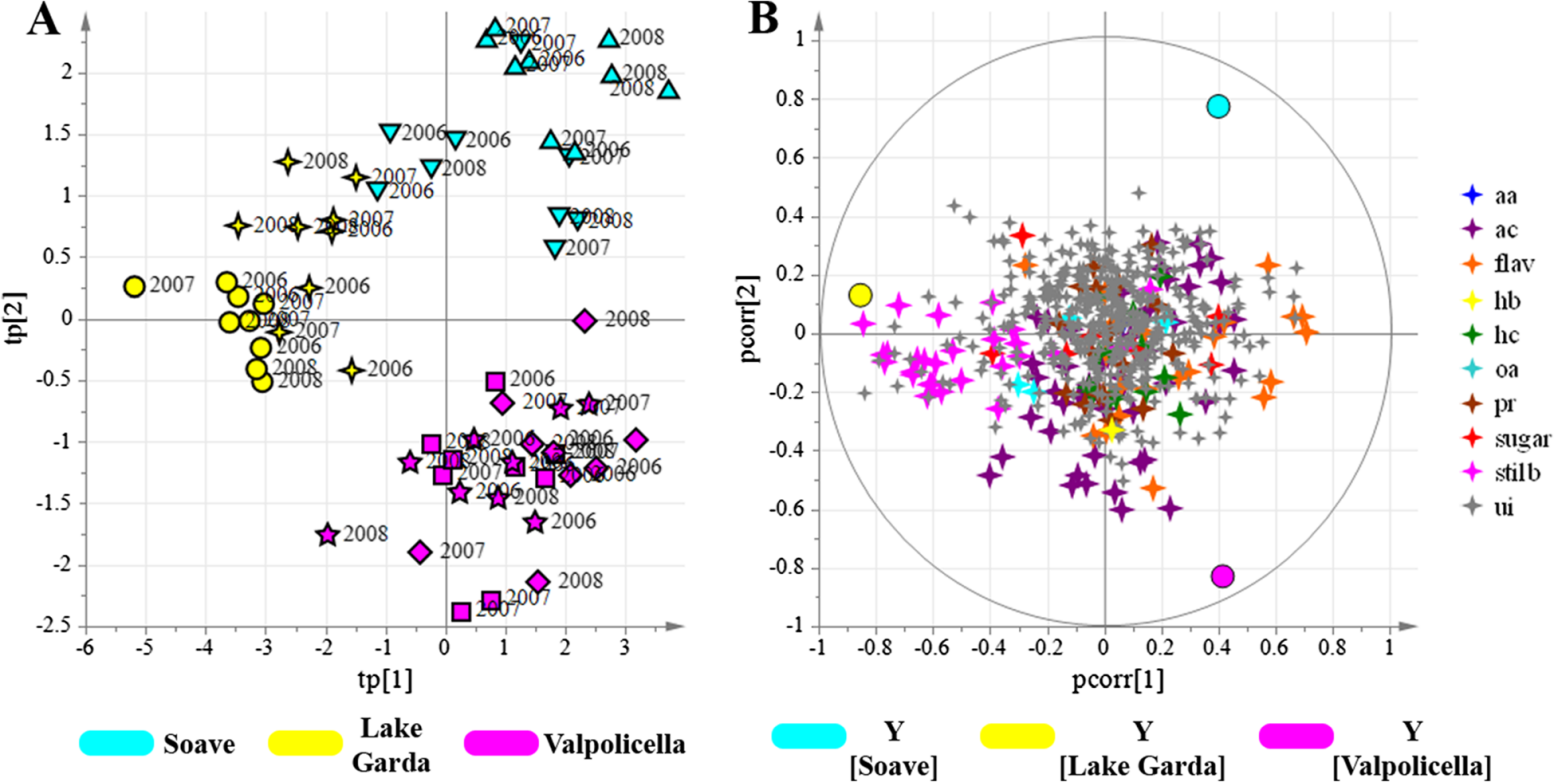
oCPLS2-DA score scatter plot (**a**) and correlation loading plot (**b**) of the model for the metabolites detected by HPLC-ESI-MS. Samples, corresponding to seven grape vineyards at three developmental stages are separated according to the geographical macrozones, regardless of the vintage. Groups of metabolites are depicted in different colors. Vineyards: ▼ = AM; ● = BA; ◼ = BM; ✦ = CS; ♦ = FA; ★ = MN; ▲ = PM. aa = amino acid; ac = anthocyanin; flav = flavonoid; hb = hydroxybenzoic acid; hc = hydroxycinnamic acid; oa = organic acid; pr = procyanidin; s = sugar; st = stilbene and viniferin; ui = unidentified

**Fig. 3:**
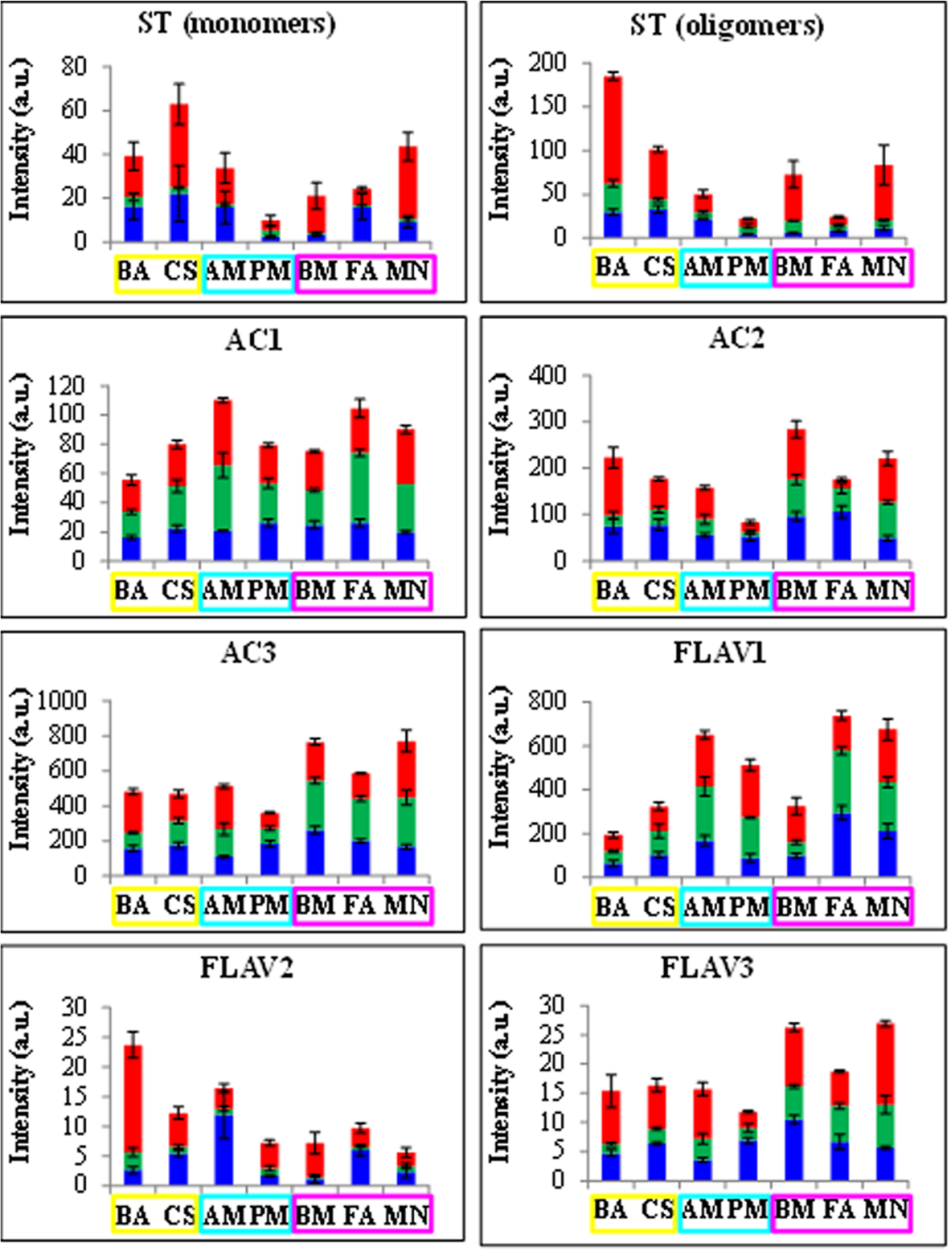
Distribution of macrozone metabolic markers, determined by HPLC-MS analysis, among the individual vineyards and in all three vintages. The markers are listed in Additional file 9: Table S6 and are assigned to a chemical class and classified according to macrozone relevance, as shown in Additional file 10: Table S7. Blue bars = 2006 vintage; green bars = 2007 vintage; red bars = 2008 vintage. Yellow rectangle: Lake Garda macrozone; sky blue: Soave macrozone; fuchsia: Valpolicella macrozone. a.u. = arbitrary units

The geographical oCPLS2-DA model for non-volatile metabolites showed four components (R^2^ = 0.79, Q^2^_6-fold CV_ = 0.71, Q^2^_7-fold CV_ = 0.73, Q^2^_8-fold CV_ = 0.71). The score scatter plot in Fig. 2a shows a clear separation of the samples from each of the three macrozones. The correlation loading plot (Fig. 2b) revealed the presence of groups of metabolites characterizing each macrozone. Specifically, stilbenes clearly characterized vineyards located in the Lake Garda macrozone, some flavonoids characterized Soave and Valpolicella vineyards, and the different vineyards and macrozones were also characterized by different anthocyanins (Additional file 9: Table S6). These differences were investigated in more detail by characterizing the putative markers of fully-mature berries listed in Additional file 9: Table S6 and assigning them to a particular chemical class (Additional file 10: Table S7). The results are shown for each of the seven vineyards in Fig. 3.

Among the stilbenes that were markers of the Lake Garda macrozone, resveratrol dimers, trimers and tetramers (ST oligomers) were particularly associated with vineyard BA. In contrast, the ST monomers resveratrol, resveratrol glucoside (piceide) and piceatannol glucoside (astringin) were not identified as general markers of the Lake Garda macrozone and were not associated with vineyard BA, but they were positively correlated with the other Lake Garda vineyard, CS.

Among the anthocyanin markers, some Valpolicella and Soave vineyards were characterized by acylated anthocyanins (AC1), whereas Lake Garda and Valpolicella vineyards were characterized by some non-acylated anthocyanins (AC2), and other Valpolicella vineyards were strongly characterized by other non-acylated anthocyanins (AC3, especially the more decorated molecules delphinidin and petunidin). Among the flavonoid markers, some quercetin derivatives characterized the Valpolicella and Soave vineyards (FLAV1), one taxifolin derivative mainly characterized the Lake Garda vineyards (FLAV2), and another putative flavanone characterized the Valpolicella vineyards (FLAV3).

Other common flavonoids, such as myricetin glycosides and various flavanones (dihydrokaempferol and naringenin glycosides) did not strongly characterize any of the vineyards under investigation. Furthermore, the flavan-3-ols, procyanidins and phenolic acid derivatives did not strongly correlate with any of the samples under investigation, with the exception of a hydroxytyrosol derivative that negatively correlated with the Lake Garda vineyards. This indicated substantial differences between distinct classes of secondary metabolites in terms of their ability to respond to *terroir*-specific environmental stimuli.

**Fig. 4:**
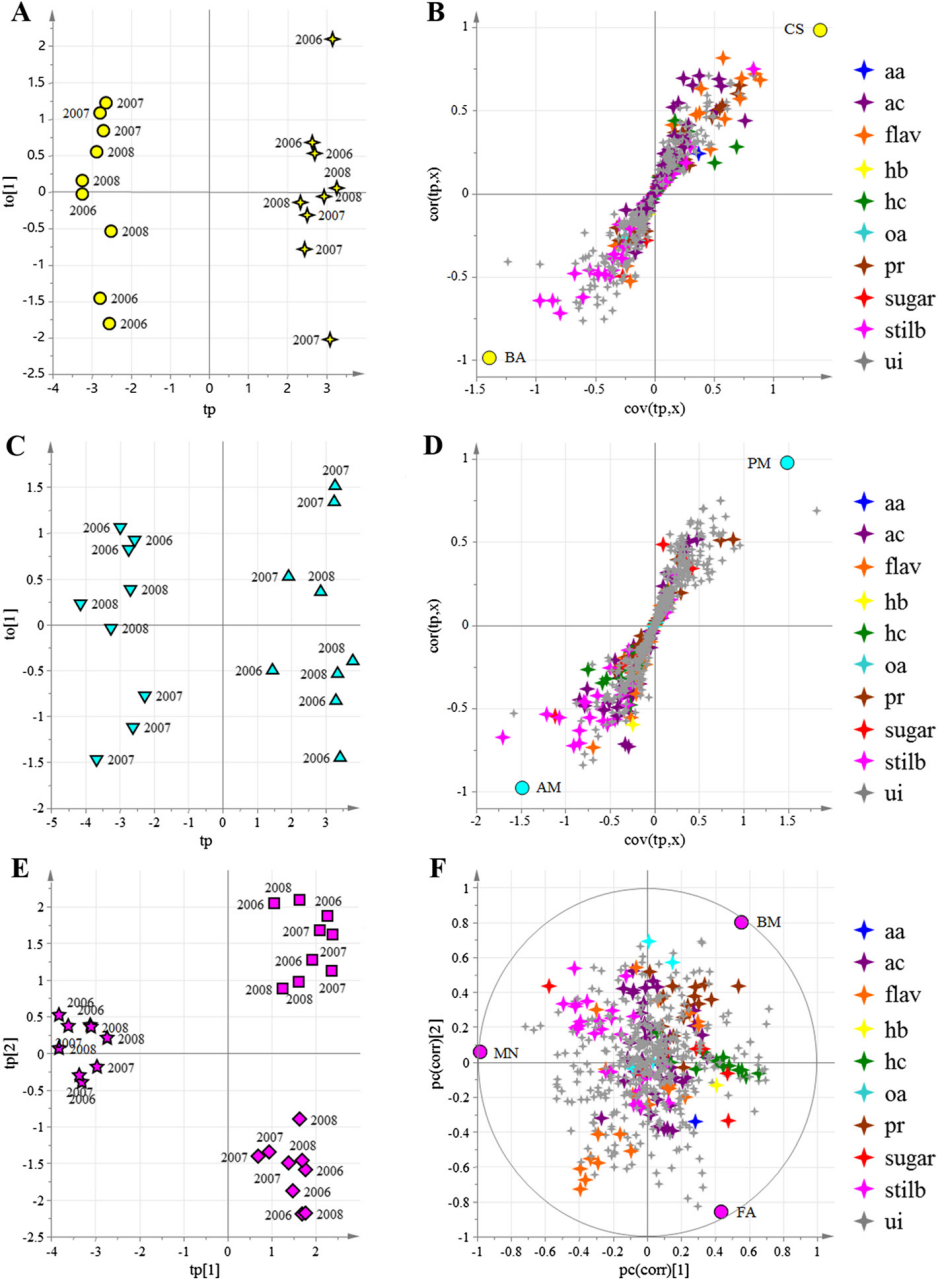
oCPLS2-DA models using the metabolites detected by HPLC-ESI-MS applied within each of the three geographical regions to distinguish the vineyards. For each model, the score scatter plot (**a**, **c**, **e**) and correlation loading plot (**b**, **d**, **f**) are provided. Samples, corresponding to seven vineyards at three developmental stages are separated regardless of the vintage. Vineyards: ▼ = AM; ● = BA; ◼ = BM; ✦ = CS; ♦ = FA; ★ = MN; ▲ = PM. Yellow (**a**, **b**): Lake Garda macrozone; sky blue (**c**, **d**): Soave macrozone; fuchsia (**e**, **f**): Valpolicella macrozone. Groups of metabolites are shown in different colors. aa = amino acid; ac = anthocyanin; flav = flavonoid; hb = hydroxybenzoic acid; hc = hydroxycinnamic acid; oa = organic acid; pr = procyanidin; s = sugar; st = stilbene and viniferin; ui = unidentified

In the second data analysis step, oCPLS2-DA was applied in each of the three geographical regions and the models showed that the producers were clearly separated from each other (Fig. 4 and Additional file 11: Table S8). The resulting model for Lake Garda had two components (R^2^ = 0.97, Q^2^_6-fold CV_ = 0.89, Q^2^_7-fold CV_ = 0.92, Q^2^_8-fold CV_ = 0.91), the model for Valpolicella had three components (R^2^ = 0.95, Q^2^_6-fold CV_ = 0.93, Q^2^_7-fold CV_ = 0.92, Q^2^_8-fold CV_ = 0.92) and the model for Soave had two components (R^2^ = 0.95, Q^2^_6-fold CV_ = 0.91, Q^2^_7-fold CV_ = 0.91, Q^2^_8-fold CV_ = 0.92).

The two vineyards in the Lake Garda macrozone were characterized by the abundance of stilbenes (BA) and some anthocyanins and flavonoids (CS). Within the Soave macrozone, vineyard AM was characterized by certain stilbenes, anthocyanins and flavonoids, whereas vineyard PM was characterized predominantly by unidentified metabolites. The three Valpolicella vineyards could be distinguished based on the content of flavan-3-ols and procyanidins (BM), coumarated malvidin (FA) and certain stilbenes (MN).

**Fig. 5:**
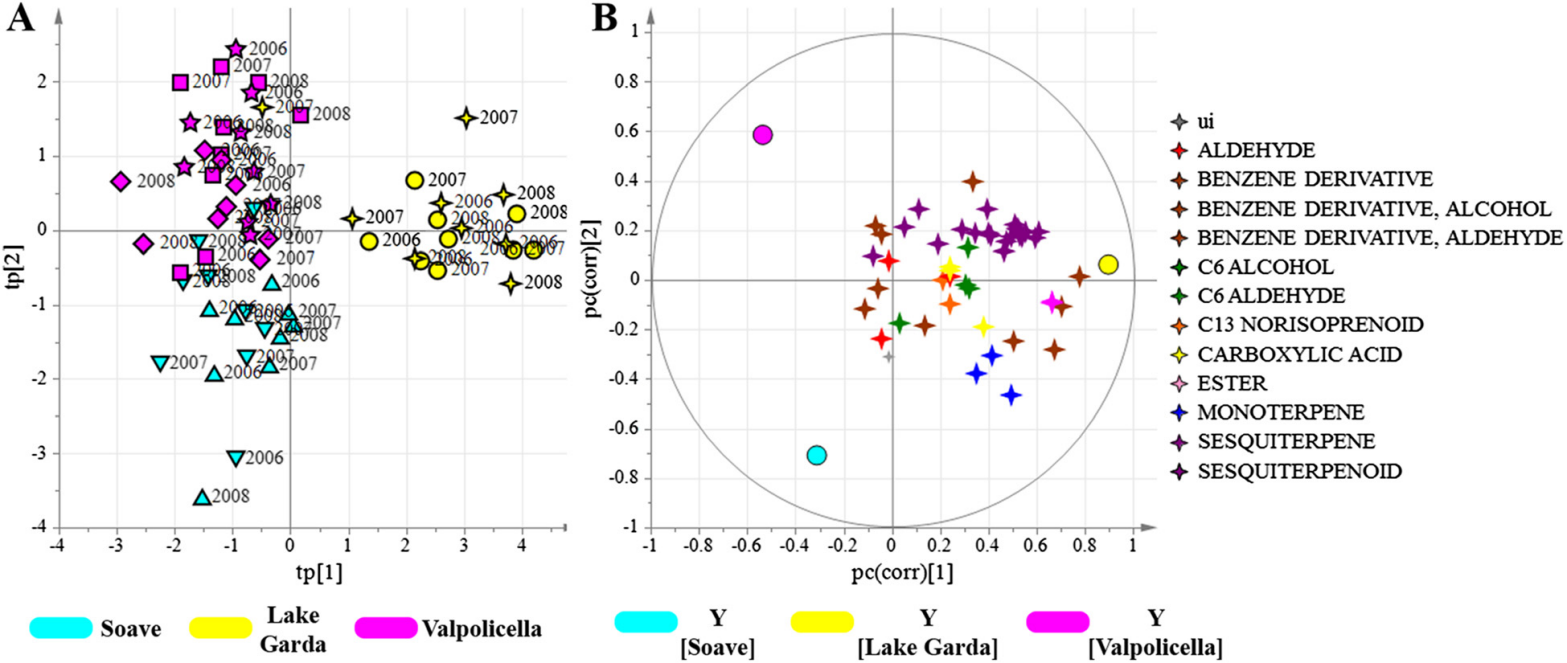
oCPLS2-DA score plot (**a**) and correlation loading plot (**b**) using the volatile metabolites as *X* variables. Samples, corresponding to seven vineyards at three developmental stages are separated according to the geographical macrozones, regardless of the vintage. Groups of metabolites are shown in different colors. ui = unidentified. Vineyards: ▼ = AM; ● = BA; ◼ = BM; ✦ = CS; ♦ = FA; ★ = MN; ▲ = PM

**Fig. 6:**
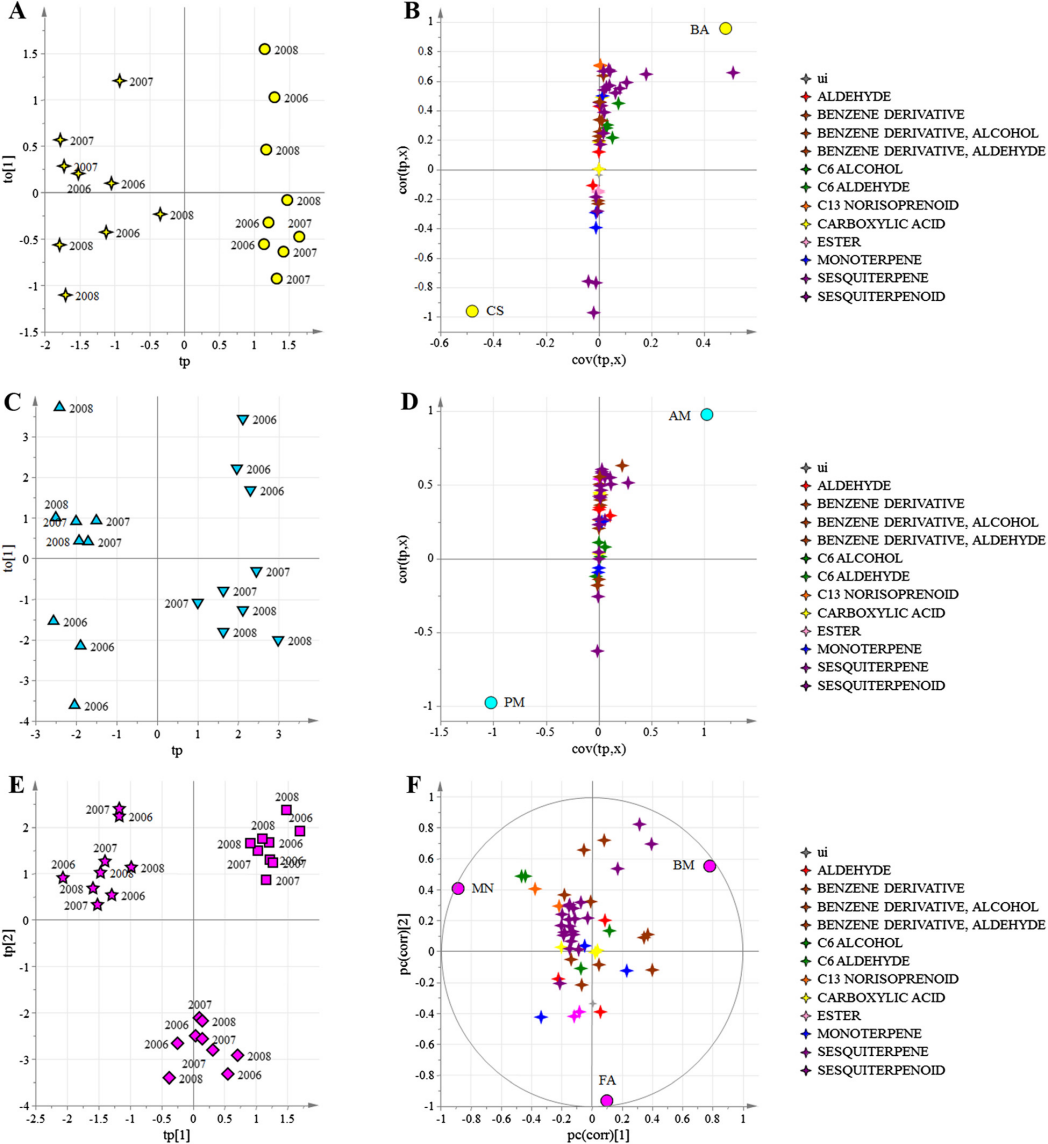
oCPLS2-DA models using the volatile metabolites applied within each of the three geographical regions to distinguish the vineyards. For each model, the score scatter plot (**a**, **c**, **e**) and the correlation loading plot (**b**, **d**, **f**) are provided. Samples, corresponding to seven vineyards at three developmental stages are separated regardless of the vintage. Vineyards: ▼ = AM; ● = BA; ◼ = BM; ✦ = CS; ♦ = FA; ★ = MN; ▲ = PM. Yellow: Lake Garda macrozone (**a**, **b**); sky blue: Soave macrozone (**c**, **d**); fuchsia: Valpolicella macrozone (**e**, **f**). Groups of metabolites are shown in different colors. ui = unidentified

The same oCPLS2-DA strategy was applied to the volatile metabolites detected by GC-MS. Once again, we were able to distinguish the three macrozones and each of the vineyards within each macrozone. The oCPLS2-DA model for geographical origin revealed five components (R^2^ = 0.68, Q^2^_6-fold CV_ = 0.46, Q^2^_7-fold CV_ = 0.49, Q^2^_8-fold CV_ = 0.45) whereas the model for the Lake Garda producers had one component (R^2^ = 0.92, Q^2^_6-fold CV_ = 0.89, Q^2^_7-fold CV_ = 0.91, Q^2^_8-fold CV_ = 0.90), the model for the Valpolicella producers had five components (R^2^ = 0.93, Q^2^_6-fold CV_ = 0.76, Q^2^_7-fold CV_ = 0.80, Q^2^_8-fold CV_ = 0.79) and the model for the Soave producers had three components (R^2^ = 0.95, Q^2^_6-fold CV_ = 0.80, Q^2^_7-fold CV_ = 0.80, Q^2^_8-fold CV_ = 0.82) as shown in Figs. 5a and 6. The Lake Garda vineyards were best characterized by this approach, on the basis of benzene derivatives, esters, sesquiterpenes and monoterpenes (Fig. 5b). Vineyard BA was mainly characterized by sesquiterpenes and C13 norisoprenoids, whereas vineyard CS was characterized by certain sesquiterpenes (Fig. 6a, b and Additional file 12: Table S9). In the Soave macrozone, vineyard AM was characterized by benzene derivatives, esters and several sesquiterpenes (Fig. 6c, d). Finally, in the Valpolicella macrozone, vineyard MN was characterized by C6 aldehydes and C13-norisoprenoids, whereas vineyard FA was characterized by low levels of benzene derivatives and some sesquiterpenes (Fig. 6e, f).

### Berry transcriptome analysis supports environment-dependent metabolome plasticity

In order to investigate the environment-dependent plasticity of some components of the Corvina metabolome, we retrieved berry transcriptomic data from the seven wine vineyards sampled in the 2008 growing season (BA, CS, BM, MN, FA, AM and PM) from our previous work [14] in which we reported the general plasticity of the entire grapevine berry transcriptome using the same biological material described herein. First, we inspected the expression profiles of the *Vitis vinifera* stilbene synthase gene family [19] throughout our experimental design. Stilbene synthases are key enzymes catalyzing the final step in the phenylalanine/polymalonate branch of the phenylpropanoid pathway that eventually produces stilbenes. The heat map shows the clear upregulation of most of the family starting from the mid-ripening stage in berries from vineyards BA and CS (Lake Garda) and pronounced upregulation in fully-mature berries from vineyards BM and MN, in line with the metabolomic data (Fig. 3). We then analyzed the expression profiles of the laccase gene family (Additional file 13: Figure S2), one member of which (*transparent testa 10, tt10*) is involved in the oxidative polymerization of phenolic compounds in the *Arabidopsis thaliana* phenylpropanoid pathway [20]. Analysis using LacSubPred software [21] showed that the laccases expressed after véraison were mainly class 8 enzymes like *tt10*, and were expressed differentially in berries from vineyards BA and CS, which are characterized by stilbenes with different degrees of polymerization (Figs. 2 and 3).

**Fig. 7:**
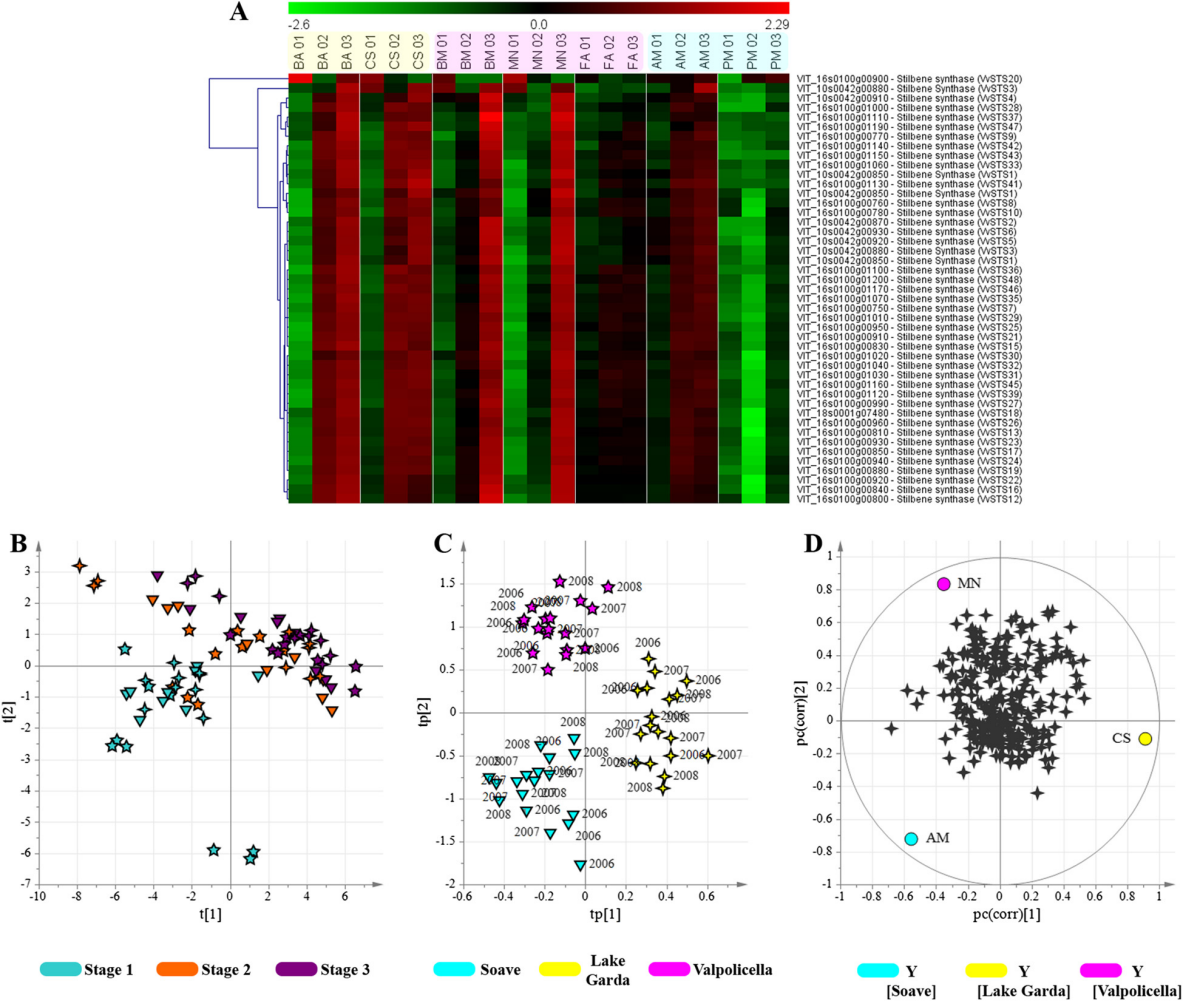
Grapevine berry transcriptome analysis. Heat map of the stilbene synthase gene family (VvSTSs) showing transcriptional profiles (**a**). The heat map was generated with TMeV v4.8.1 using the average expression level of the three replicates. Data were normalized based on the mean center genes/rows adjustment, and Pearson’s correlation was chosen as the statistical metric. PCA score scatter plot obtained using transcripts related to secondary metabolism (**b**; explained variance equal to 69 %). Stage 1: beginning of véraison; stage 2: pre-ripening; stage 3: full maturity. oCPLS2-DA score scatter plot (**c**) and correlation loading plot (**d**). Samples are separated according to the geographical macrozones, regardless of the vintage. Vineyards: ▼ = AM; ✦ = CS; ★ = MN. In (**d**) the circles represent the macrozone, while the ✦ symbols represent the transcripts

The statistical approach described above was used to retrieve transcripts associated with the geographical area regardless of the vintage. This was achieved by creating a data set containing berry transcriptomic data representing all three developmental stages of each vintage, sourced from three vineyards, one representing each macrozone (CS from Lake Garda, MN from Valpolicella and AM from Soave). The data set included 292 selected genes involved in non-volatile secondary metabolism (Additional file 14: Table S10). Based on PCA results (Fig. 7b), we applied oCPLS2-DA to both the mid ripening and fully mature berries, because the accumulation of a metabolite in fully mature fruits is often triggered by an earlier transcriptional change (Fig. 7c, d). The score scatter plot and the correlation loading plot of the obtained model (four components, R^2^ = 0.83, Q^2^_6-fold CV_ = 0.70, Q^2^_7-fold CV_ = 0.77, Q^2^_8-fold CV_ = 0.73) are reported in Fig. 7c and d, respectively. Vineyard MN, which is associated with the positive metabolomic markers AC1, AC3, FLAV1 and FLAV3 (Fig. 3), was also found to be associated with transcripts for the three transcription factors VvMybA1, VvMybA2 and VvMybA3 (VIT_02s0033g00410, VIT_02s0033g00380 and VIT_02s0033g00450, respectively), a flavonoid 3',5'-hydroxylase (VIT_06s0009g02910) and a 4-coumarate-CoA ligase (VIT_17s0000g01790) (Additional file 14: Table S10), all of which are active in the berry anthocyanin biosynthesis pathway [22]. This vineyard was also associated with transcripts for two flavonol synthases (VIT_13s0047g00210, VIT_07s0031g00100) and the transcription factor VvMybF1 (VIT_07s0005g01210), which are involved in berry flavonol synthesis [23], again supporting the metabolomic data. Similarly, vineyard CS, which is characterized at the metabolomic level by the abundance of stilbenes (Fig. 3), was found to be associated with transcripts for the R2-R3 MYB transcription factor VvMYB14 (VIT_07s0005g03340) (Additional file 14: Table S10) which regulates berry stilbene biosynthesis [24]. Interestingly, Soave vineyard AM lacked strongly positive transcriptomic markers, but was associated with several negative transcriptomic markers linked to the low level of AC2 anthocyanins (Fig. 3), including anthocyanin O-methyltransferase VvAOMT1 (VIT_01s0010g03510), MATE efflux family protein VvAnthoMATE2 (VIT_16s0050g00910), UDP glucose:flavonoid 3-o-glucosyltransferase VvUFGT (VIT_16s0039g02230) and anthocyanin membrane protein 1 (Anm1, VIT_08s0007G03560). We also observed a correlation between the low level of FLAV1 molecules in berries from vineyard CS and the presence among its negative markers of VvMyb5a (VIT_08s0007g07230), a transcription factor involved in the general grapevine flavonoid pathway [25, 26]. When the same statistical approach was applied to a data set of selected volatile-related transcripts, we found no correlation among the transcripts and volatile metabolites (data not shown).

### Correlation between secondary metabolites and specific *terroir* features

We investigated the specific responses of Corvina berries to terroir-specific environmental components by generating several classifications for each of the components described in Additional file 1: Table S1; only some of these classifications resulted in O2PLS-DA models, reported in Table 1, correlating the berry metabolome with the vineyard features (see Additional file 1: Table S1). We considered as reliable only those models with a Q^2^_7-fold CV_ value greater than 0.5 that also passed the permutation test on the response (400 random permutations). Table 1 shows a complete list of the models we tested and their relative Q^2^_7-fold CV_ values, with the reliable models highlighted in bold.

**Table 1:** List of the OPLS-DA models that were validated using a cross-validation test with 200 permutations, showing the classes that were used

		Non-volatile metabolome	Volatile metabolome
OPLS-DA model	Classes	Q^2^	Q^2^
**Macrozone**	Soave vs. valpolicella vs. lake garda	**0.541**	0.384
**Macrozone**	Lake garda vineyards vs. others	**0.635**	**0.536**
**Vineyard altitude**	>200 m vs. < 200 m	**0.556**	0.0117
Row direction	N–S vs. E–W	0.396	**0.521**
Training system	Parral vs. guyot	-	**0.549**
**Soil type**	pH (<8 vs. >8)	**0.631**	**0.621**
**Soil type**	Total lime % (<10 vs. 10–20 vs. >20)	**0.671**	-
Soil type	Active lime % (<4 vs. > 4)	0.179	**0.646**
Soil type	Loam % (<30 vs. 30–50 vs. >50)	0.322	0.29
**Soil type**	Clay % (<25 % vs. >25 %)	**0.582**	0.226
Soil type	Sand % (<20 vs. 20–50 vs. >50)	0.198	-
**Soil type**	Organic matter % (<2 vs. 2–2.5 vs. >2.5)	**0.588**	0.368
Soil type	Exchangeable phosphorus mg/kg (<30 vs. 30–50 vs. >50)	0.174	-
**Soil type**	Exchangeable potassium mg/kg (<200 vs. 200–400 vs. >400)	**0.669**	0.426
Soil type	Exchangeable magnesium mg/kg (<300 vs. 300–700 vs. >700)	0.446	0.426
Soil type	Exchangeable calcium mg/kg (3000–8000 vs. >8000)	0.291	0.086

Fully-validated models with a Q^2^ value greater than 0.5 are shown in bold separately for the non-volatile (LC-MS) and volatile (GC-MS) data sets

The loading plot showed that no individual metabolites correlated strongly (pq(corr) >0.75) with any of the *terroir* features. Even in the best models for metabolites detected by LC-MS (macrozone, Lake Garda vs. others, soil total lime, soil clay, soil exchangeable potassium) and by GC-MS (macrozone, Lake Garda vs. others, training system and soil active lime) there was only a low correlation between individual metabolites and specific *terroir* features (Additional file 15). The existence of reliable O2PLS-DA models lacking strong characteristic metabolites suggests that the observed correlations between specific *terroir* features and the berry metabolome probably reflect many small metabolomic changes rather than a small number of major metabolic shifts. In the context of these slight correlations, we found once again that flavonoids and stilbenes were assigned to the more plastic fraction of the metabolome, given that some flavonoids correlated with the Valpolicella and Soave macrozones, low soil clay, total lime and exchangeable potassium, whereas stilbenes correlated with the Lake Garda macrozone, low soil clay and an average amount of soil exchangeable potassium.

## Discussion

The complex relationship between the composition of grape berries and the environment of the grapevine plant during ripening was investigated by untargeted metabolomics analysis (LC-MS and GC-MS), transcriptomics analysis (microarray expression profiles) and the development and application of appropriate chemometric statistical analysis methods. We studied a single clone of the Corvina variety to eliminate any genotype-dependent variability, thus focusing solely on responses to the *terroir*. The variability of the responses therefore depended entirely on the plasticity of the selected genotype.

In our recent analysis of the berry transcriptome, we observed a strong vintage-specific effect on gene expression [14]. As anticipated, we also observed such an effect on the metabolome in the current study, suggesting that *terroir*-specific effects can only be determined by including multiple vintages in the analysis, to avoid confusing *terroir*-specific effects with differences caused by the growing season. For example, favorable growing seasons such as 2008 resulted in the accumulation of most of the secondary metabolites we detected, whereas a small number of unidentified metabolites were more characteristic of the less favorable 2007 season. The climate is probably the most important vintage-specific factor affecting berry quality at harvest. A minimum cumulative temperature (expressed as the Huglin heat summation index) must be achieved during the growing season to ensure the complete ripening of certain cultivars [27, 28]. Temperatures that are too low delay ripening, but temperatures that are too high promote early ripening which also reduces the berry quality. The 2007 vintage had a relatively high Huglin index with a harvest date set at the end of August. No anthocyanins correlated with in the 2007 vintage, probably reflecting the damaging effect of solar radiation on the berries, and the fact that temperatures exceeding 35 °C inhibit color development [29, 30]. Stilbenes and viniferins were also less abundant in the 2007 vintage, consistent with previous findings that stilbene and viniferin levels decline in dry seasons [31]. The 2008 vintage was positively correlated with anthocyanins and flavanones, whereas the 2006 vintage showed a composition that was intermediate between 2007 and 2008. The 2008 and 2006 vintages were rated as very good or outstanding [32, 33] whereas the 2007 vintage was rated as good.

The non-volatile metabolites detected by LC-MS responded mainly to the ripening program, although some responded both to the ripening program and environmental parameters. Hydroxycinnamic acid derivatives and flavan-3-ols/procyanidins appeared to correlate mainly with the ripening program, with the levels of both declining from véraison to full ripeness. In the full-mature berries they proved to be the less plastic components of the metabolome, since their did not characterize any of the vineyards, thus showing poor ability to respond to the different environmental solicitations. Other components of the metabolome, including flavonoids, stilbenes and anthocyanins, were strongly dependent on the developmental program but nevertheless showed significant plasticity with respect to the seven individual vineyards and the three different macrozones. High levels of stilbenes and low levels of flavonoids were typical of the two vineyards located in the Lake Garda macrozone, whereas certain flavonoids were characteristic of the vineyards in the Soave and Valpolicella macrozones, and different anthocyanins characterized the different vineyards and macrozones. Within each of the macrozones, the individual vineyards were easily distinguishable by their specific chemical signatures.

In terms of *terroir*-specific features, correlations were observed mainly for stilbenes and flavonoids. Previous studies have considered the environment-dependent accumulation of secondary metabolites in ripened berries but have focused on individual factors or small groups, typically including light, water and temperature [10, 11, 34–36]. Strong light induces the expression of genes representing the flavonoid and anthocyanin biosynthesis pathways, and the accumulation of both metabolites (which protect plants from excess solar irradiation), whereas shading alters the composition of anthocyanins and reduces the accumulation of flavonols [37, 38]. Water deficit induces the accumulation of anthocyanins and has a variable effect on stilbenes and flavononols [39–43]. Low temperatures induce the accumulation of anthocyanins whereas high temperatures inhibit the accumulation of both anthocyanins and flavonoids [44, 45]. More complex environmental parameters such as the elevation of vineyards have also been considered, and stilbenes tend to accumulate at higher elevations, albeit in a cultivar-dependent manner [46].

The *terroir* in which a vine grows and its berries ripen is more complex than the individual factors described above because multiple factors combine and interact to generate a large number of variables. At least three different types of environmental variable may contribute to the *terroir*, namely the vintage (e.g., climatic factors), stable environmental features (e.g., soil composition and viticultural practices) and variables reflecting the interaction between vintage-specific factors and stable environmental features. The experimental approaches used during this investigation allowed us to separate the vintage-specific effects from those caused by more stable environmental features, whereas effects potentially caused by the interaction between these components were not revealed by our analysis. Our approach was able to remove the effects caused by vintage, thus highlighting groups of metabolites characterizing each geographical macrozone and each vineyard within a macrozone, providing a reliable objective benchmark for the concept of *terroir*.

We investigated the relationship between stable *terroir*-specific features and the metabolic profile of the berries in detail. The composition of berries is known to be affected by soil properties [47–51] and viticultural practices [52–55]. We found that the vineyard altitude and several soil properties (pH, total lime, active lime, percentage clay, organic matter and exchangeable potassium) correlated with the composition of non-volatile metabolites. We also found that viticultural practices (row direction and training system) and certain soil properties (pH and active lime) correlated with the composition of volatile metabolites. Despite these results, the Q^2^ values were low and no individual metabolite achieved a pq(corr) value greater than 0.75 for any of the *terroir* features. However, we observed correlations for broad categories of metabolites (e.g., active lime and volatile metabolites, and vineyard altitude and non-volatile metabolites). These data indicate that the observed clustering reflected small correlated changes in many metabolites rather than radical changes in the levels of a few key metabolites.

Transcriptomics analysis revealed that the accumulation of several metabolites induced by *terroir*-specific environmental conditions was positively correlated with the regulation of the corresponding metabolic pathways at the level of transcription. The clearest example was the stilbene synthase gene family in the Lake Garda macrozone, and to a lesser extent also in the Valpolicella macrozone (Fig. 7a). Previous studies have shown that the expression of genes related to stilbene synthesis is enhanced by environmental stress, especially water deficit [56], wounding, UV-C exposure and infection with pathogens [19]. These data suggest that the environmental and viticultural parameters characterizing the Lake Garda macrozone may act as modulators of stilbene metabolism during the ripening of Corvina berries.

Genes related to anthocyanin and flavonoid synthesis also appeared to be influenced by the *terroir* and their expression showed a positive correlation with the accumulation of the corresponding metabolites. This suggests that the *terroir* may induce a climate-independent change in the composition of phenolic compounds by transcriptome remodeling that persists over different vintages. Previous reports have indicated that the berry-specific expression of genes related to anthocyanin synthesis shows substantial plasticity and is greatly influenced by the environment [9, 44, 57].

Finally, we did not find a clear correlation between the accumulation of volatile metabolites and the expression of genes required for their synthesis, suggesting that the plasticity of the volatile metabolome might not be solely controlled by regulating the transcription of genes involved in the corresponding biosynthesis pathways. Indeed, volatiles found in grapes might be formed by still unknown pathways or they might origin from non-biological reactions.

## Conclusions

The metabolome and transcriptome characterization of grape berries from a single clone of the Corvina variety cultivated in seven different vineyards, over a three-year trial period, together with the development of statistical tools to overcome the dominant vintage effects, allowed us to see a terroir-dependent plasticity of the metabolome and of the related transcripts, which persists over several vintages. Within the various metabolite classes, clear differences in the terroir dependent plasticity were seen: stilbenes, anthocyanins, flavonoids and some VOCs (especially sesquiterpenes) proved to be the more plastic component of the metabolome, while other component, such as the procyanindins and flavan-3-ols were much more stable. On the other side, only weak relationship were observed between the metabolome and individual terroir-specific features (including soil composition and viticultural practices).

## Availability of supporting data

The data sets supporting the metabolome results of this article are included within the article and its additional files. The microarray data were downloaded from Gene Expression Omnibus (GEO) using accession number GSE41633 at website http://www.ncbi.nlm.nih.gov/geo/query/acc.cgi?acc=GSE41633.

## Additional files

Additional file 1: Table S1. Principal features of each vineyard: a) macrozone; b) height above sea level; c) rootstock type; d) row direction (N–S = north–south; E–W = east–west; e) training system; f) soil type, based on USDA classification triangle (www.nrcs.usda.gov/Internet/FSE_DOCUMENTS/16/stelprdb1044821.ppt); g) total lime content, percentage; h) active lime content, percentage; i) pH; j) organic substance content, percentage; k) exchangeable phosphorus content (mg/kg). Soil composition data were provided by ORVIT – Società per la Valorizzazione dei Vini Veronesi (Verona, Italy). (DOCX 20 kb) Additional file 2: Table S2. Main climatic parameters recorded for the 2006, 2007 and 2008 vintages by meteorological stations located in each macrozone (Lake Garda, Valpolicella and Soave) and kindly provided by A.R.P.A.V. (Azienda Regionale per la Prevenzione Ambientale del Veneto, Centro Metereologico di Teolo, Padova, Italy). The values reported for each parameter relate to: a) the period 1 April to 30 September and 2) ripening phenological stage (42 days before harvesting). The Huglin index is defined as the sum of average and maximum temperatures above 10 °C in the period 1 April to 30 September for a given location; K = 1.04. (DOCX 16 kb) Additional file 3:
**Heat map representing the areas of the main chromatographic peaks assessed by HPLC-DAD and HPLC-ESI-MS for the seven vineyards and fully-ripened berries.** Each value represents the average of the three replicates. Areas assessed with HPLC-DAD were measured at 320 nm for Hc, 520 nm for Ac and 290 nm for Fl. The values for LC-ESI-MS samples are the same reported in the data matrix obtained after processing with MZmine. Rt: retention time, *m/z*: mass/charge ratio; Hc: hydroxycinnamic acid; Ac: anthocyanin; Fl: flavonoid; AM, BA, BM, CS, FA, MN, PM represent the vineyards. (DOCX 390 kb) Additional file 4:
**Method used to build the orthogonal constrained PLS-DA model and the mathematical properties of the approach used to post-transform the oCPLS2-DA model.** (DOCX 134 kb) Additional file 5: Table S3. List of molecules identified in the HPLC-ESI-MS data set generated by MZmine software. a) ID based on MZmine data matrix; b) row *m/z* of parent ions; c) retention time (RT) in minutes; d) putative identification; e) class; aa: amino acids; ac: anthocyanins; flav: flavonoids; fr: fragment; hb: hydroxybenzoic acids; hc: hydroxycinnamic acids; oa: organic acids; pr: procyanidins; s: sugars; st: stilbenes; t: tannins; ui: unidentified; f) MS/MS fragments of parent ions; fragments selected for further fragmentation are shown in bold; g) MS^3^fragments generated from ions reported in bold in the MS/MS column. (XLSX 42 kb) Additional file 6: Table S4. List of molecules identified in the GC-MS data set generated by Agilent Chemstation software. a) ID generated by Agilent Chemstation software; b) retention time (RT) in minutes; c) Kovats retention Index (KI); d) putative identification; e) class; f) typical descriptors. (XLSX 14 kb) Additional file 7: Table S5. Lists of the volatile and non-volatile annotated metabolites, classified according to the ANOVA analysis. The metabolites that significantly varied according to the vintage or the year (p-value < 0.01) are indicated as “1”, while the other as “0”. (XLSX 28 kb) Additional file 8: Figure S1. PLS-DA score plot (A) and correlation loading plot (B) of the HPLC-ESI-MS data set for samples classified according to ripening stage. Stage 1: beginning of véraison; stage 2: pre-ripening; stage 3: full maturity stage. PLS-DA score plot (C) and correlation loading plot (D) of the HPLC-ESI-MS data set for samples classified according to growing season. Blue: 2006; green: 2007; red: 2008. PLS-DA score plot (E) and correlation loading plot (F) of the GC-MS data set for samples classified according to growing season. Blue: 2006; green: 2007; red: 2008. Vineyards: ▼ = AM; ● = BA; ◼ = BM; ✦ = CS; ♦ = FA; ★ = MN; ▲ = PM. Groups of metabolites are shown in different colors. ui = unidentified. (TIFF 2617 kb) Additional file 9: Table S6. List of markers in the non-volatile metabolite data set for the three geographical macrozones. a) ID based on the data set in Additional file 5: Table S3; b) raw *m/z* of parent ion; c) retention time (RT) in minutes; d) putative identification; e) class; aa: amino acids; ac: anthocyanins; flav: flavonoids; fr: fragment; hb: hydroxybenzoic acids; hc: hydroxycinnamic acids; oa: organic acids; pr: procyanidins; s: sugars; st: stilbenes; ui: unidentified; f) marker type: molecules found at low levels (<) and high levels (>) in the selected macrozone are indicated; g) pcorr [1]; h) pcorr [2]. (XLSX 21 kb) Additional file 10: Table S7. Macrozone-specific markers in the non-volatile metabolite data set classified according to their chemical class and macrozone relevance; the classification is the same as used in Fig. 3. (XLSX 135 kb) Additional file 11: Table S8. List of non-volatile markers for the seven vineyards, divided by macrozone; a) Lake Garda (vineyards BA and CS); b) Valpolicella (vineyards BM, FA and MN); c) Soave (vineyards AM and PM). Each data set shows: a) ID based on the data set in Additional file 5: Table S3; b) raw *m/z* of parent ion; c) retention time (RT) in minutes; d) putative identification; e) class; aa: amino acids; ac: anthocyanins; flav: flavonoids; fr: fragment; hb: hydroxybenzoic acids; hc: hydroxycinnamic acids; oa: organic acids; pr: procyanidins; s: sugars; st: stilbenes; t: tannins; ui: unidentified; f) marker type: molecules found at low levels (<) and high levels (>) in the selected macrozone are indicated; g) cor (tp, x) values; h) cov (tp, x) values. (XLSX 163 kb) Additional file 12: Figure S2. Heat map of the laccase gene family, showing transcriptional profiles. The heat map was generated with TMeV v4.8.1 using the average expression level of three replicates. Data were normalized based on the mean center genes/rows adjustment and Pearson’s correlation was chosen as a statistical metric. The legend indicates the enzyme class predicted by LacSubPred software as described by Weirick et al. [21]. (XLSX 27 kb) Additional file 13: Table S9. List of the markers for the volatile metabolite data set for the three geographical macrozones and the seven vineyards within each macrozone. a) Lake Garda (vineyards BA and CS); b) Valpolicella (vineyards BM, FA and MN); c) Soave (vineyards AM and PM). (JPEG 115 kb) Additional file 14: Table S10. Transcript data sets related to non-volatile and volatile markers of the different vineyards according to the oCPLS2-DA method. Microarray fluorescence intensities were downloaded from Gene Expression Omnibus (GEO) using accession number GSE41633. Negative (<) and positive (>) markers for each vineyard are indicated. (XLSX 328 kb) Additional file 15.
**Loading plots of several O2PLS-DA models investigating the relationships between environmental and vineyard features and metabolites detected by HPLC-ESI-MS and GC-MS.** Each feature and the relative statistical classes are indicated in the plot. Groups of metabolites are shown in different colors according to the legends. (PDF 394 kb)

## Abbreviations

LC-MS: Liquid chromatography-mass spectrometry
HPLC-DAD: High performance liquid chromatography-diode array detector
HPLC-ESI-MS: High performance liquid chromatography-electrospray ionization-mass spectrometry
GC-MS: Gas chromatography-mass spectrometry
PCA: Principal component analysis
PLS-DA: Partial least squares projection to latent structures discriminant analysis
O2PLS-DA: Orthogonal projection to latent structures discriminant analysis
oCPLS2-DA: Orthogonal constrained partial least squares projection to latent structures discriminant analysis
