# Supplementary material for: Towards a scientific interpretation of the terroir concept: plasticity of the grape berry metabolome
Source: BMC Plant Biol. 2015 Aug 7;15:191. doi: 10.1186/s12870-015-0584-4 (PMC4527360; doi:10.1186/s12870-015-0584-4)
Supplement: Additional file 1: Table S1. — Principal features of each vineyard: a) macrozone; b) height above sea level; c) rootstock type; d) row direction (N–S = north–south; E–W = east–west; e) training system; f) soil type, based on USDA classification triangle (www.nrcs.usda.gov/Internet/FSE_DOCUMENTS/16/stelprdb1044821.ppt); g) total lime content, percentage; h) active lime content, percentage; i) pH; j) organic substance content, percentage; k) exchangeable phosphorus content (mg/kg). Soil composition data were provided by ORVIT – Società per la Valorizzazione dei Vini Veronesi (Verona, Italy). (DOCX 20 kb) [file 12870_2015_584_MOESM1_ESM.docx]

|  | **AM** | **BA** | **BM** | **CS** | **FA** | **MN** | **PM** |
| --- | --- | --- | --- | --- | --- | --- | --- |
| **Macrozone** | Soave | Lake Garda | Valpolicella | Lake Garda | Valpolicella | Valpolicella | Soave |
| **Height (m)** | 250 | 120 | 450 | 100 | 130 | 250 | 130 |
| **Rootstock** | 41B | S04 | K5BB | 420A | 420A | K5BB | 41B |
| **Row direction** | E-W | N-S | E-W | E-W | E-W | N-S | N-S |
| **Training system** | Parral | Parral | Guyot | Parral | Parral | Guyot | Guyot |
| **Soil type** | Silty clay | Loam | Clay | Loam | Clay Loam | Silt loam | Clay loam |
| **Total lime %** | 3.9 | 19.3 | 18.3 | 14.4 | 31 | 5.9 | 27.9 |
| **Active lime %** | 0.5 | 2.6 | 9.4 | 6.3 | 11.3 | 3.1 | 8.3 |
| **Sand %** | 15 | 47 | 66 | 42 | 29 | 13 | 36 |
| **Loam %** | 43 | 36 | 21 | 37 | 39 | 67 | 36 |
| **Clay %** | 42 | 17 | 13 | 21 | 32 | 20 | 28 |
| **Soil pH** | 8.3 | 7.9 | 7.8 | 8.2 | 8.2 | 7.8 | 7.9 |
| **Organic substance (%)** | 2.9 | 2.5 | 2.2 | 1.2 | 2.9 | 1.6 | 2.5 |
| **Exchangeable phosphorus (mg/kg)** | 26 | 73 | 73 | 68 | 48 | 47 | 64 |
| **Exchangeable potassium (mg/kg)** | 190 | 376 | 620 | 230 | 168 | 154 | 126 |
| **Exchangeable magnesium (mg/kg)** | 272 | 468 | 848 | 623 | 294 | 293 | 183 |
| **Exchangeable calcium (mg/kg)** | 6500 | 5380 | 7358 | 6346 | 4652 | 10055 | 2878 |
| **Berry Reducing Sugars 2006** | 211.25 ± 1.20 | 176.20 ± 0.42 | 187.40 ± 0.00 | 203.70 ± 1.13 | 212.55 ± 0.64 | 195.20 ± 0.00 | 211.65 ± 0.64 |
| **Berry Reducing Sugars 2007** | 190.00 ± 1.27 | 165.25 ± 0.49 | 153.00 ± 0.42 | 203.60 ± 0.71 | 210.90 ± 0.71 | 192.25 ± 0.64 | 188.70 ± 1.84 |
| **Berry Reducing Sugars 2008** | 191.35 ± 0.64 | 178.90 ± 0.57 | 170.05 ± 0.49 | 205.15 ± 1.48 | 188.70 ± 0.57 | 169.35± 0.49 | 108.05 ± 1.06 |
| **Berry pH 2006** | 3.01 ± 0.01 | 2.96 ± 0.01 | 2.84 ± 0.00 | 2.9 ± 0.00 | 2.98 ± 0.00 | 3.02 ± 0.00 | 3.06 ± 0.01 |
| **Berry pH 2007** | 2.97 ± 0.00 | 3.00 ± 0.00 | 2.74 ± 0.00 | 3.07 ± 0.01 | 2.98 ± 0.00 | 2.87 ± 0.01 | 3.09 ± 0.00 |
| **Berry pH 2008** | 2.83 ± 0.00 | 3.04 ± 0.01 | 2.71 ± 0.00 | 2.98 ± 0.01 | 2.98 ± 0.00 | 2.82 ± 0.00 | 3.11 ± 0.00 |

**Additional file 1: Table S1**
