# Supplementary material for: Towards a scientific interpretation of the terroir concept: plasticity of the grape berry metabolome
Source: BMC Plant Biol. 2015 Aug 7;15:191. doi: 10.1186/s12870-015-0584-4 (PMC4527360; doi:10.1186/s12870-015-0584-4)
Supplement: Additional file 2: Table S2. — Main climatic parameters recorded for the 2006, 2007 and 2008 vintages by meteorological stations located in each macrozone (Lake Garda, Valpolicella and Soave) and kindly provided by A.R.P.A.V. (Azienda Regionale per la Prevenzione Ambientale del Veneto, Centro Metereologico di Teolo, Padova, Italy). The values reported for each parameter relate to: a) the period 1 April to 30 September and 2) ripening phenological stage (42 days before harvesting). The Huglin index is defined as the sum of average and maximum temperatures above 10 °C in the period 1 April to 30 September for a given location; K = 1.04. (DOCX 16 kb) [file 12870_2015_584_MOESM2_ESM.docx]

**Additional file 2: Table S2**

|  | **Lake Garda** | | | **Valpolicella** | | | **Soave** | | |
| --- | --- | --- | --- | --- | --- | --- | --- | --- | --- |
|  | **2006** | **2007** | **2008** | **2006** | **2007** | **2008** | **2006** | **2007** | **2008** |
| total rainfall (mm)  (Apr-Sept) | 453.8 | 327.2 | 580.6 | 552.8 | 495.8 | 629.2 | 604.2 | 296.2 | 521.4 |
| total rainfall (mm)  (ripening) | 220.0 | 51.2 | 167.8 | 340.2 | 104.8 | 171.6 | 235.0 | 54.0 | 94.0 |
| % rainfall in ripening | 48.5 | 15.6 | 28.9 | 61.5 | 21.1 | 27.3 | 38.9 | 18.2 | 18.0 |
| Huglin Index  (Apr-Sept) | 2432.8 | 2571.7 | 2410.8 | 2239.7 | 2344.9 | 2149.4 | 2458.5 | 2625.0 | 2439.5 |
| Huglin Index  (ripening) | 590.9 | 729.1 | 605.6 | 541.6 | 682.2 | 567.0 | 585.3 | 754.9 | 635.0 |
| average temp.  (Apr-Sept) | 19.0 | 19.3 | 19.1 | 18.7 | 18.7 | 18.2 | 19.3 | 19.6 | 19.1 |
| average temp.  (ripening) | 20.4 | 23.4 | 21.2 | 19.9 | 22.9 | 20.4 | 20.3 | 24.0 | 21.3 |
| T min/max  (Apr-Sept) | 1.6 36.8 | 5.3 37.1 | 2.5 34.2 | 3.2 35.2 | 7.3 35.2 | 5.8 32.6 | 1.4 37.0 | 6.9 37.9 | 2.9 35.3 |
| T min/max  (ripening) | 8.9 32.2 | 11.8 37.1 | 6.8 32.9 | 10.7 30.3 | 12.4 35.2 | 8.1 30.4 | 10.1 31.7 | 13.1 37.9 | 7.7 33.0 |
| average humidity  (Apr-Sept) | 68.1 | 67.0 | 72.4 | 61.0 | 59.5 | 65.2 | 60.5 | 58.1 | 64.2 |
| average humidity  (ripening) | 72.1 | 64.9 | 72.8 | 66.0 | 57.4 | 63.2 | 66.0 | 56.5 | 62.6 |
