# Supplementary figures and images for: Towards a scientific interpretation of the terroir concept: plasticity of the grape berry metabolome
Source: BMC Plant Biol. 2015 Aug 7;15:191. doi: 10.1186/s12870-015-0584-4 (PMC4527360; doi:10.1186/s12870-015-0584-4)

**
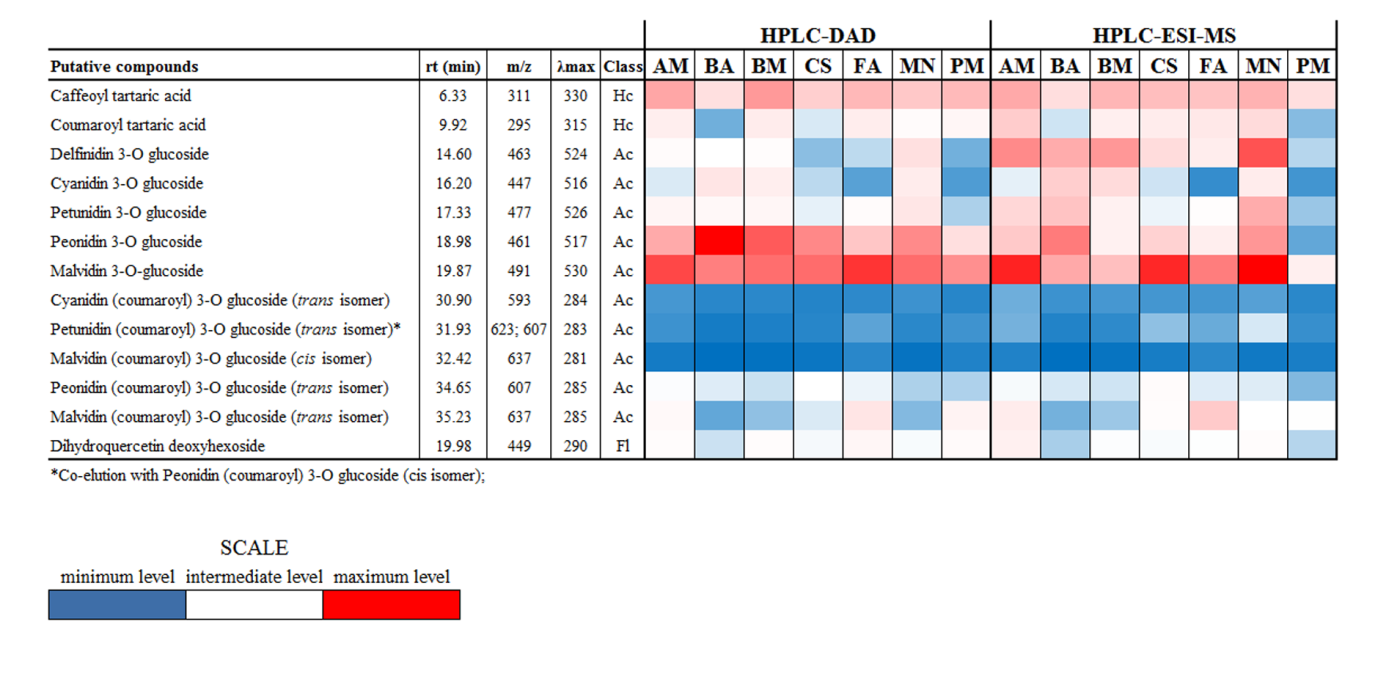
Additional file 3**

Supplement: Additional file 3: — Heat map representing the areas of the main chromatographic peaks assessed by HPLC-DAD and HPLC-ESI-MS for the seven vineyards and fully-ripened berries. Each value represents the average of the three replicates. Areas assessed with HPLC-DAD were measured at 320 nm for Hc, 520 nm for Ac and 290 nm for Fl. The values for LC-ESI-MS samples are the same reported in the data matrix obtained after processing with MZmine. Rt: retention time, m/z: mass/charge ratio; Hc: hydroxycinnamic acid; Ac: anthocyanin; Fl: flavonoid; AM, BA, BM, CS, FA, MN, PM represent the vineyards. (DOCX 390 kb) [file 12870_2015_584_MOESM3_ESM.docx]

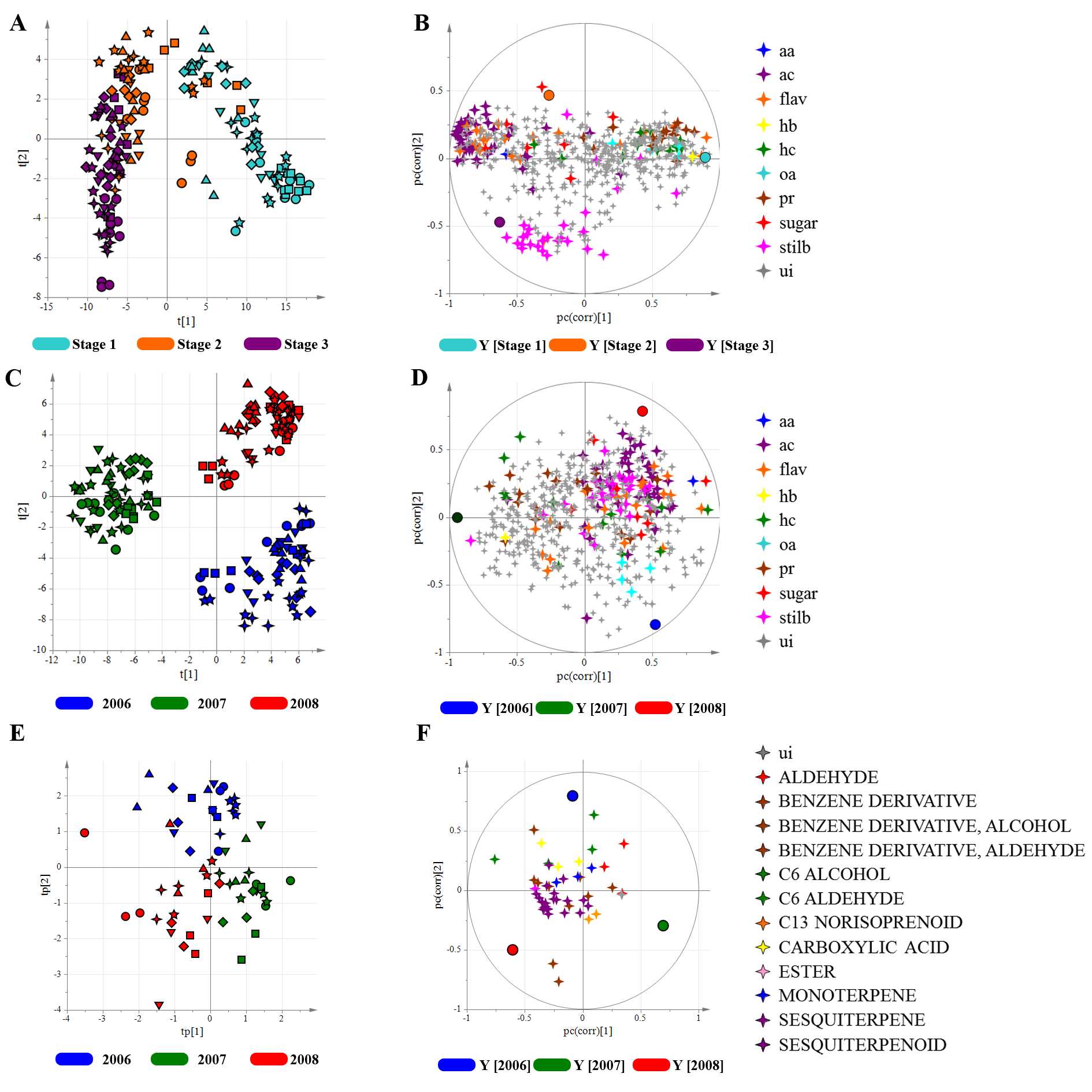

Supplement: Additional file 8: Figure S1. — PLS-DA score plot (A) and correlation loading plot (B) of the HPLC-ESI-MS data set for samples classified according to ripening stage. Stage 1: beginning of véraison; stage 2: pre-ripening; stage 3: full maturity stage. PLS-DA score plot (C) and correlation loading plot (D) of the HPLC-ESI-MS data set for samples classified according to growing season. Blue: 2006; green: 2007; red: 2008. PLS-DA score plot (E) and correlation loading plot (F) of the GC-MS data set for samples classified according to growing season. Blue: 2006; green: 2007; red: 2008. Vineyards: ▼ = AM; ● = BA; ◼ = BM; ✦ = CS; ♦ = FA; ★ = MN; ▲ = PM. Groups of metabolites are shown in different colors. ui = unidentified. (TIFF 2617 kb) [file 12870_2015_584_MOESM8_ESM.tif]

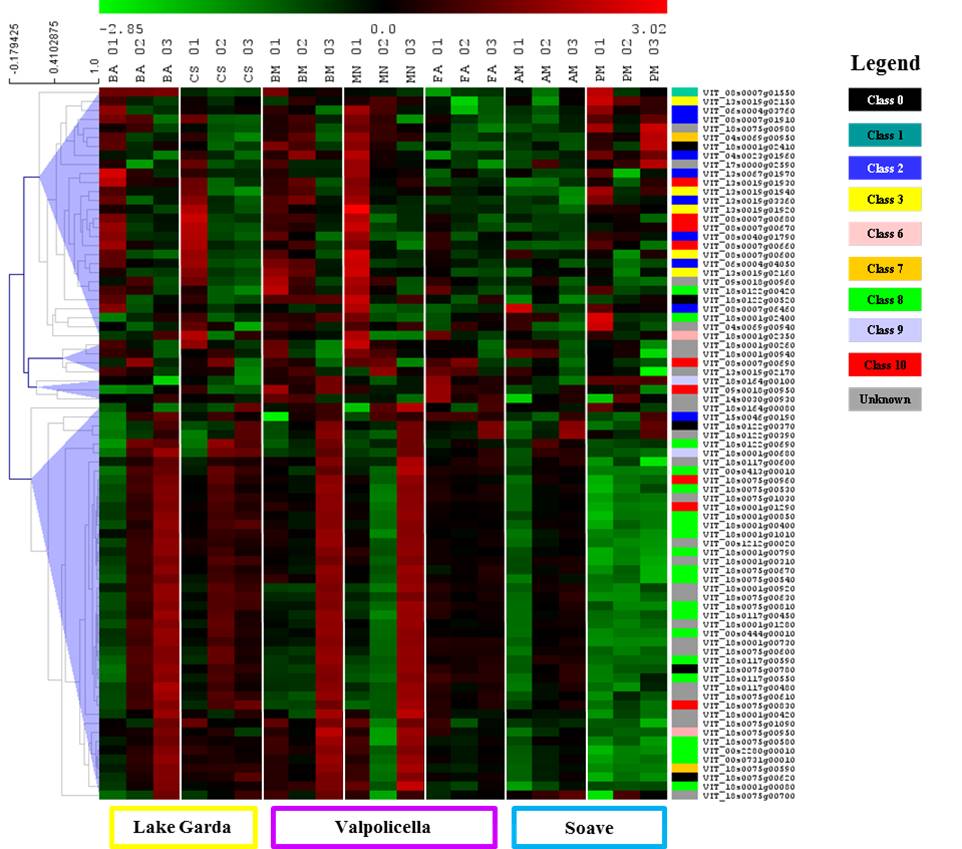

Supplement: Additional file 13: Table S9. — List of the markers for the volatile metabolite data set for the three geographical macrozones and the seven vineyards within each macrozone. a) Lake Garda (vineyards BA and CS); b) Valpolicella (vineyards BM, FA and MN); c) Soave (vineyards AM and PM). (JPEG 115 kb) [file 12870_2015_584_MOESM13_ESM.jpg]
