# Supplementary material for: Towards a scientific interpretation of the terroir concept: plasticity of the grape berry metabolome
Source: BMC Plant Biol. 2015 Aug 7;15:191. doi: 10.1186/s12870-015-0584-4 (PMC4527360; doi:10.1186/s12870-015-0584-4)
Supplement: Additional file 4: — Method used to build the orthogonal constrained PLS-DA model and the mathematical properties of the approach used to post-transform the oCPLS2-DA model. (DOCX 134 kb) [file 12870_2015_584_MOESM4_ESM.docx]

**Additional file 4**

**PROJECTION TO LATENT STRUCTURES (PLS) TECHNIQUES**

In this Supplementary file we introduce the method applied to build the orthogonal Constrained PLS-DA model and the mathematical properties of the approach used to post-transform the oCPLS2-DA model.

Following Wold’s approach, we can split the PLS algorithm (here we use PLS to indicate the PLS2 algorithm) into two main parts: one where the weight vector $\text{w}_{i}$for projecting the residual matrix $E_{i-1}$ of the X-block is calculated by solving

$\text{w}_{i}:\mathop{\mathrm{argmax}}\limits_{\text{w}_{i}^{t}\text{w}_{i}=1} \text{w}_{i}^{t}\left(E_{i-1}^{t}F_{i-1}F_{i-1}^{t}E_{i-1}\right) \text{w}{}_{i}$ S1

and the other corresponding to an iterative algorithm where the residuals of the X-block and those of the Y-block $F_{i-1}$ (this is not necessary if the scores of the Y-block are not required) are projected onto the space orthogonal to the score vector $\text{t}_{i}$ calculated by using $\text{w}_{i}$. Given the two data matrices $X$ and $Y$, the PLS algorithm can be summarized as

$$E_{0}\leftarrow X$$

$$F_{0}\leftarrow Y$$

*i* = 1

1 solve $\left(E_{i-1}^{t}F_{i-1}\right)\left(E_{i-1}^{t}F_{i-1}\right)^{t}\text{w}_{i}=s_{i}^{2} \text{w}_{i}$

$$\text{t}_{i}=E_{i-1}\text{w}_{i}$$

$$E_{i}=\hat{Q}_{\text{t}_{i}}E_{i-1}$$

$$F_{i}=\hat{Q}_{\text{t}_{i}}F_{i-1}$$

*i* = *i* + 1 go to 1 for other components

where $\hat{Q}_{\text{t}_{i}}=I-\text{t}_{i}\text{t}_{i}^{\text{t}}/\left(\text{t}_{i}^{t}\text{t}_{i}\right)$ is an orthogonal projection matrix able to project any vector onto the space orthogonal to $\text{t}_{i}$, $I$ is the identity matrix and step 1 is the solution of S1. It is possible to obtain the regression matrix $B_{\text{PLS2}}$ which is used to calculate the modeled response matrix$\hat{Y}=XB_{\text{PLS2}}$ on the basis of the weight matrix $W$ by

$B_{\text{PLS2}}=W\left(W^{t}X^{t}XW\right)^{-1}W^{t}X^{t}Y$.

The PLS algorithm can be modified to include orthogonal constraints for the weight vector $\text{w}_{i}$ as shown below. By considering a matrix $Z$, we want to calculate a weight vector $\text{w}_{i}$ that can project the X-block following the framework of the PLS algorithm but under the constraint $Z\text{w}_{i}=0$. In this respect, the maximization problem at the iteration $i$ of the PLS algorithm can be formulated as

$\mathop{\mathrm{argmax}}\limits_{\begin{matrix} \text{w}_{i}^{t}\text{w}_{i}=1 \\ \text{c}_{i}^{t}\text{c}_{i}=1 \\ Z\text{w}_{i}=0 \end{matrix}} \text{w}_{i}^{t}E_{i-1}^{t}F_{i-1}\text{c}_{i}$ S2

where $E_{i-1}$ and $F_{i-1}$ are the residual matrices for the X- and Y-blocks, respectively, and $\text{c}_{i}$ is the weight vector for projecting the Y-block. The solution can be found by considering that the vector $\text{w}_{i}$ belongs to the kernel of $Z$ or is orthogonal to the column space of $Z$. We chose the second route by assuming

where $\hat{Q}=I-VV^{t}$ is the orthogonal projection matrix that can transform each vector $\tilde{\text{w}}_{i}$ into a vector orthogonal to $Z=U\Lambda V^{t}$. Indeed, it is possible to calculate

.

Then, the maximization problem S2 can be re-written as

.

and the solution obtained by applying the Lagrange’s multipliers method. As a result, the weight vector to use is the eigenvector corresponding to the highest eigenvalue of the problem

$H_{i}^{t}H_{i} \text{w}_{i}=s_{i}^{2}\text{w}_{i}$ S3

where $H_{i}=F_{i-1}^{t}E_{i-1}\hat{Q}$.

This result can be usefully applied to find score vectors $\text{t}_{i}$ orthogonal to the column space of a matrix $M$ for performing PLS regression. We call the method orthogonal Constrained PLS2 (oCPLS2). The maximization problem at the iteration $i$ of the iterative algorithm for PLS can be now formulated as

$\mathop{\mathrm{argmax}}\limits_{M^{t}\text{t}_{i}=0} \text{t}_{i}^{t}\text{u}_{i}=\mathop{\mathrm{argmax}}\limits_{\begin{matrix} \text{w}_{i}^{t}\text{w}_{i}=1 \\ \text{c}_{i}^{t}\text{c}_{i}=1 \\ \left(M^{t}E_{i-1}\right)\text{w}_{i}=0 \end{matrix}} \text{w}_{i}^{t}E_{i-1}^{t}F_{i-1}\text{c}_{i}$.

It can be proven that $M^{t}E_{i-1}=M^{t}X=Z$ at any iteration. As a consequence, the solution is S3 having

$$\hat{Q}=I-VV^{t}$$

where $V$ is obtained by singular value decomposition of $M^{t}X$.

The following algorithm is able to calculate the oCPLS2 model for given $X$, $Y$ and $M$matrices

$$E_{0}\leftarrow X$$

$$F_{0}\leftarrow Y$$

$$M^{t}E_{0}=U\Lambda V^{t}$$

$$\hat{Q}=I-VV^{t}$$

*i* = 1

1 solve

$$\text{t}_{i}=E_{i-1}\text{w}_{i}$$

$$E_{i}=\hat{Q}_{\text{t}_{i}}E_{i-1}$$

$$F_{i}=\hat{Q}_{\text{t}_{i}}F_{i-1}$$

*i* = *i* + 1 go to 1 for other components

where $\hat{Q}_{\text{t}_{i}}=I-\text{t}_{i}\text{t}_{i}^{\text{t}}/\left(\text{t}_{i}^{t}\text{t}_{i}\right)$. After *A* iterations, the regression model

$$Y=XB_{\text{oCPLS2}}+F_{A}$$

can be obtained by calculating the regression coefficient matrix

$B_{\text{oCPLS2}}=W\left(W^{t}X^{t}XW\right)^{-1}W^{t}X^{t}Y$.

As well as PLS, oCPLS2 can be used to drive discriminant analysis by introducing suitable dummy variables specifying the class membership of the collected samples.

To better explain the main differences between PLS-DA and oCPLS2-DA we discuss the model for the geographical discrimination of the collected samples of fully-ripened berries described by non-volatile metabolites. The design of the experiment is orthogonal but a large part of the variance of the dataset (approximately the 35% of the total variance) is related to the “vintage” effects as the PCA model highlighted (Figure 1C). If one performs PLS-DA and one considers the first two latent components of the model, the following correlation matrix with respect the three growing seasons is obtained

Y[2006] Y[2007] Y[2008]

t[1] 0.000 0.234 -0.234

t[2] -0.299 0.540 -0.241

proving that the latent structure discovered by PLS-DA is related to the “vintage” effect. In other words, PLS-DA produces latent components explaining both the differences due to the geographical origin and the effects of the growing season on the metabolome. The modeling of the same dataset by oCPLS2-DA produced latent variables uncorrelated to the “vintage” effects focusing the investigation only on the effects of the geographical origin.

Usually, projection to latent structures regression techniques produce a large number of latent components compromising a clear model interpretation. For this reason, we applied a suitable post-transformation of the oCPLS2-DA model.

The idea underlying our approach is to transform the oCPLS2 model into a new model by applying a suitable orthogonal transformation of the weight matrix that can produce regression coefficients equal to those of oCPLS2 but score vectors $\text{t}_{i}$ with a different behavior with respect to the response $Y$.

We report a general method valid both for PLS and oCPLS2 based on the property that any orthogonal matrix $G:G^{t}G=GG^{t}=I_{A}$ used to transform the weights of the model does not modify the matrix of the coefficients. Indeed, by considering the transformation $\tilde{W}=WG$ we can calculate

Then, the objective is to find an orthogonal matrix $G$ that is able to transform the weight matrix $W$ in order to produce two sets of scores: one composed of non-predictive scores orthogonal to the response (orthogonal part of the model) and the other with scores correlated to the response (parallel or predictive part of the model). In our method, the two sets of scores are produced by two different kinds of weight vectors that can be arranged into two different blocks of the matrix $G$. As a consequence, we consider an orthogonal matrix having the structure:

$$G=\left(G_{\text{o}}G_{\text{p}}\right)$$

where the columns of the block $G_{\text{o}}$ produce weight vectors $\tilde{\text{w}}_{\text{o}i}$ that are able to project out the orthogonal part of the model while the columns of the block $G_{\text{p}}$ generate weight vectors $\tilde{\text{w}}_{\text{p}i}$ associated with the predictive part of the model.

If we use $\text{g}_{\text{o}i}$ and $\text{g}_{\text{p}i}$ to indicate the columns of the block $G_{\text{o}}$ and those of the block $G_{\text{p}}$, respectively, we can calculate $\text{g}_{\text{o}i}$ as

$Y^{t}XW=USV^{t}$ S4

$\left(I_{A}-VV^{t}\right) \left(W^{t}X^{t}XW\right) \text{g}_{\text{o}i}=\lambda_{\text{o}i}\text{g}_{\text{o}i} \lambda_{\text{o}i}>0$ S5

and $\text{g}_{\text{p}i}$ by solving

$\left(I_{A}-G_{\text{o}}G_{\text{o}}^{t}\right)^{t}\left(I_{\text{A}}-G_{\text{o}}G_{\text{o}}^{t}\right) \text{g}_{\text{p}i}=\lambda_{\text{p}i}\text{g}_{\text{p}i} \lambda_{\text{p}i}>0$ S6

where $I_{A}$ is the identity matrix of size *A*, *A* is the number of components of the model, step S4 is the singular value decomposition of the matrix $Y^{t}XW$ and the combination of S4 and S5 corresponds to a direct orthogonal filter that can produce orthogonal scores $\text{t}_{\text{o}i}$ solving the problem

$$\mathop{\mathrm{argmax}}\limits_{Y^{t}\text{t}_{\text{o}i}=0} \text{t}_{\text{o}i}^{t}\text{t}_{\text{o}i}$$

having $\text{t}_{\text{o}i}=E_{i-1}\tilde{\text{w}}_{\text{o}i}$ under the condition $\tilde{W}=WG$.

Now, the weight matrix $\tilde{W}=WG$can be used to obtain the post-transformed model by using the iterative algorithm described above for PLS where the columns of $\tilde{W}$ are used as weight vectors instead of $W$ in step 1.

Then, our post-transformation method results to be a three steps approach: in the first step, a PLS or oCPLS regression model is built on the data; in the second step, the weight matrix of the model is transformed by the orthogonal matrix $G$ calculated by S4,S5 and S6 while in the third step a regression model is rebuilt by using the same framework of the PLS algorithm but the new weight matrix to project the data.

The relationships between the X-block and the Y-block can be investigated by exploring only the parallel part of the model by using suitable correlation loading plots or the so called w*c plot. As a result, the model obtained by post-transformation of PLS maintains the same power in prediction and regression coefficients of the untransformed PLS model but can be easily interpreted because the number of components useful to interpret the model is reduced.

An important property of our method is that the score vector $\text{t}_{\text{o}i}$ obtained by $\text{g}_{\text{o}i}$ becomes orthogonal to $Y$. Indeed, we can calculate

where we used the equality $Y^{t}E_{i-1}W=Y^{t}XW$ for the orthogonal part of the model.

The same method can be apply to oCPLS2 with the result that the new weight matrix $\tilde{W}$ satisfies the constraint $Z\tilde{W}=ZWG=0$.

An interesting application is to PLS-DA or to oCPLS2-DA, where the transformation of the weight matrix can be used to simplify model interpretation. If the problem having N classes is well-defined, $G_{\text{o}}$ produces A-N+1 orthogonal score vectors whereas $G_{\text{p}}$ generates N-1 predictive score vectors. Then, the resulting post-transformed model has only N-1 predictive components that must be investigated while the predictive power is the same of the untransformed model where the number of components was A. Then, the number of components used in model interpretation can be substantially reduced.

The R-function for post-transforming the PLS or the oCPLS2 model can be required to the corresponding author.
