# Supplementary material for: Towards a scientific interpretation of the terroir concept: plasticity of the grape berry metabolome
Source: BMC Plant Biol. 2015 Aug 7;15:191. doi: 10.1186/s12870-015-0584-4 (PMC4527360; doi:10.1186/s12870-015-0584-4)

Additional file 15 - O2PLS-DA loading plots, HPLC-MS-detected metabolites

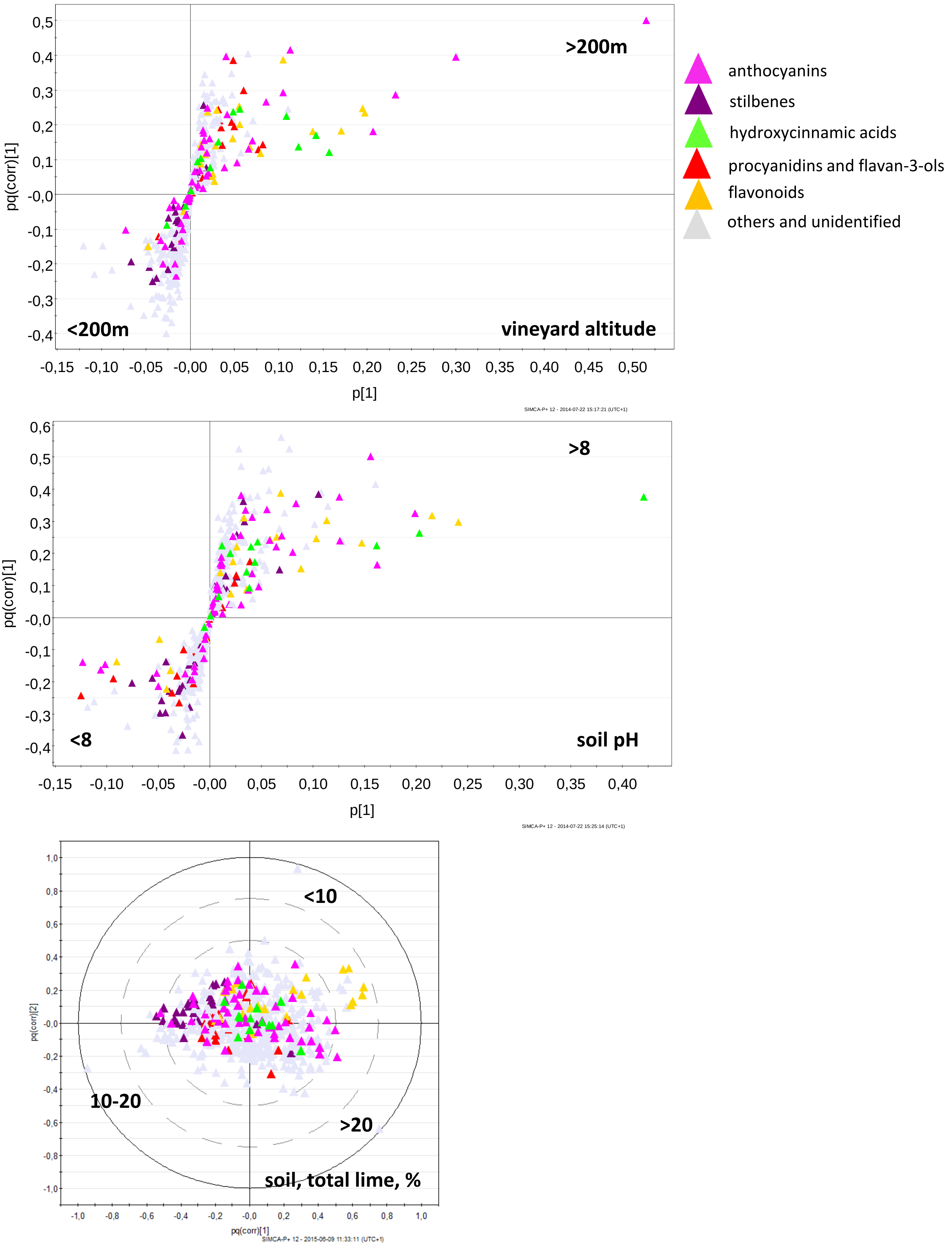

O2PLS-DA loading plots, HPLC-MS-detected metabolites

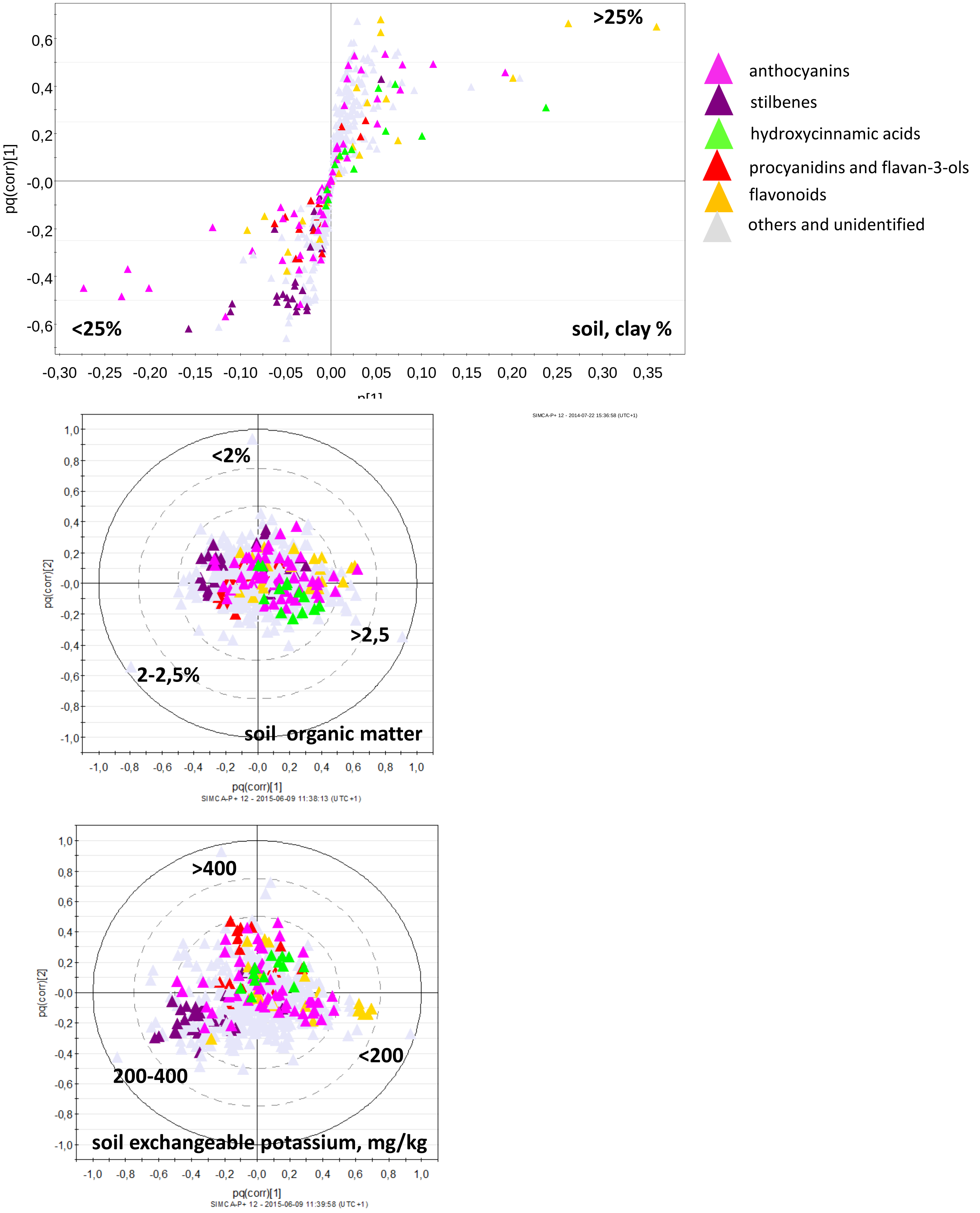

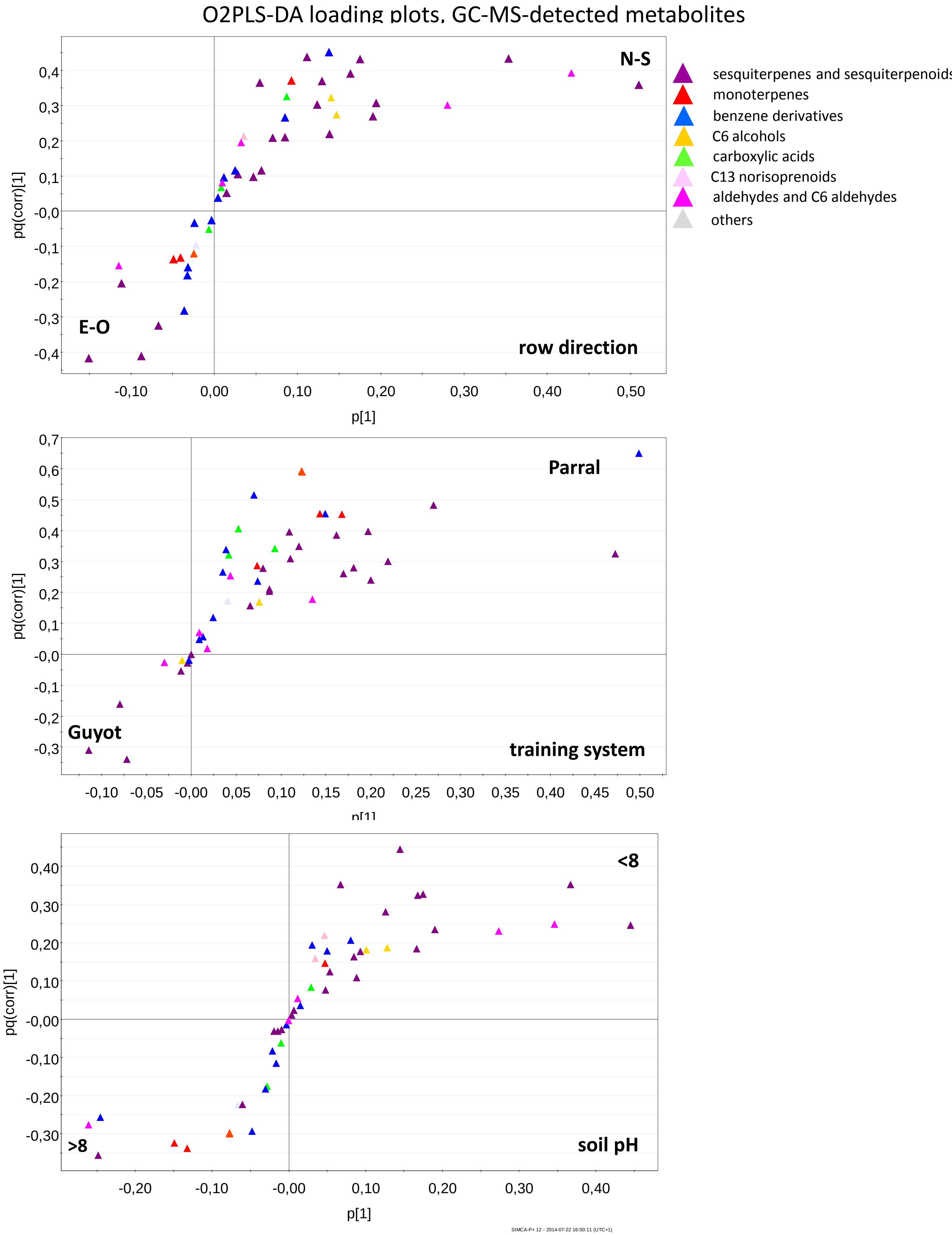

O2PLS-DA loading plots, GC-MS-detected metabolites
N-S
E-O
row direction
pq(corr)[1]
p[1]
sesquiterpenes and sesquiterpenoids
monoterpenes
benzene derivatives
C6 alcohols
carboxylic acids
C13 norisoprenoids
aldehydes and C6 aldehydes
others
Parral
Guyot
training system
<8
>8
soil pH
SIMCA-P+ 12 - 2014-07-22 16:00:11 (UTC+1)

O2PLS-DA loading plots, GC-MS-detected metabolites

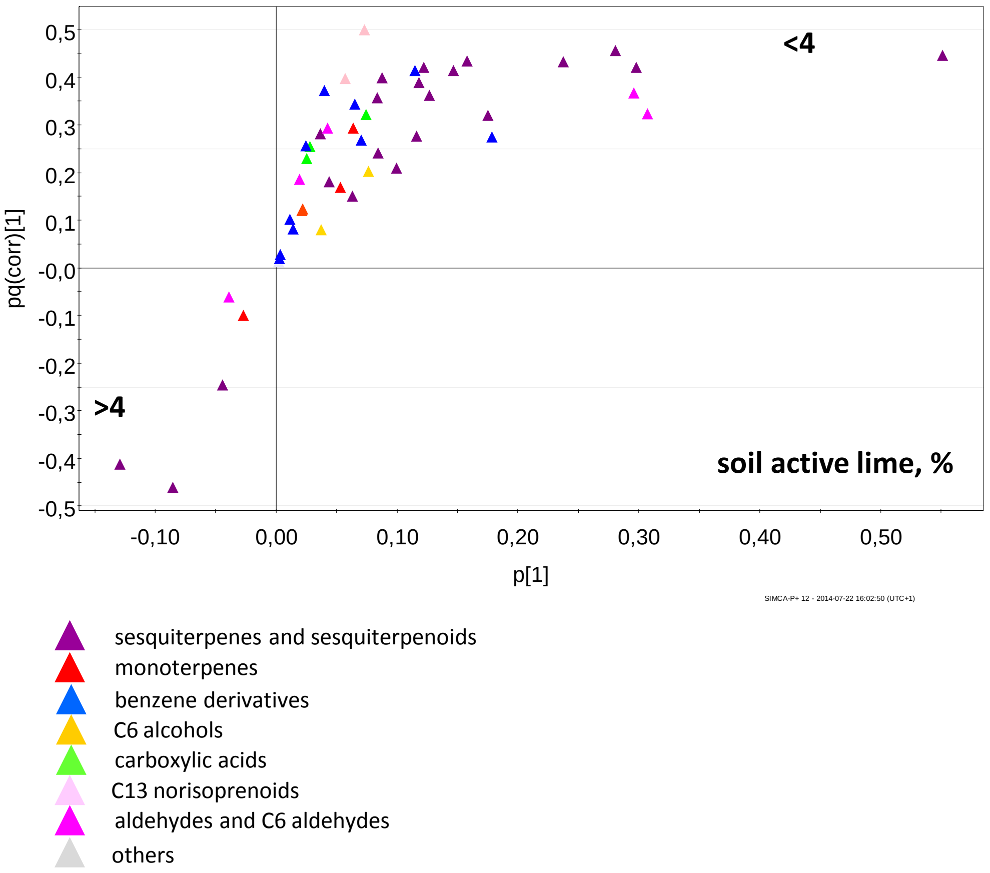

Supplement: Additional file 15. — Loading plots of several O2PLS-DA models investigating the relationships between environmental and vineyard features and metabolites detected by HPLC-ESI-MS and GC-MS. Each feature and the relative statistical classes are indicated in the plot. Groups of metabolites are shown in different colors according to the legends. (PDF 394 kb) [file 12870_2015_584_MOESM15_ESM.pdf]
